# scDALI: modeling allelic heterogeneity in single cells reveals context-specific genetic regulation

**DOI:** 10.1186/s13059-021-02593-8

**Published:** 2022-01-06

**Authors:** Tobias Heinen, Stefano Secchia, James P. Reddington, Bingqing Zhao, Eileen E. M. Furlong, Oliver Stegle

**Affiliations:** 1 Division of Computational Genomics and Systems Genetics, German Cancer Research Center (DKFZ), Heidelberg, Germany; 2 Genome Biology Unit, European Molecular Biology Laboratory, Heidelberg, Germany; 3 Faculty of Mathematics and Computer Science, Heidelberg University, Heidelberg, Germany; 4 Faculty of Biosciences, Collaboration for Joint PhD Degree between EMBL and Heidelberg University, Heidelberg, Germany

**Keywords:** Single-cell, Regulatory genomics, Statistical methods

## Abstract

While it is established that the functional impact of genetic variation can vary across cell types and states, capturing this diversity remains challenging. Current studies using bulk sequencing either ignore this heterogeneity or use sorted cell populations, reducing discovery and explanatory power. Here, we develop scDALI, a versatile computational framework that integrates information on cellular states with allelic quantifications of single-cell sequencing data to characterize cell-state-specific genetic effects. We apply scDALI to scATAC-seq profiles from developing F1 *Drosophila* embryos and scRNA-seq from differentiating human iPSCs, uncovering heterogeneous genetic effects in specific lineages, developmental stages, or cell types.

**Supplementary Information:**

The online version contains supplementary material available at 10.1186/s13059-021-02593-8.

## Background

The functional impact of genetic variants on molecular traits such as gene expression can be influenced by the cell type or cell state. Particularly non-coding variants in enhancer elements can impact a gene’s expression in one tissue and not in others. Population-scale genetics studies, using bulk sequencing across individuals, have identified many such tissue-specific [1–3] and developmental stage-specific [4] effects, which often involve rare genetic variants. However, even carefully dissected tissues are composed of heterogeneous cell types, thus motivating the application of single-cell sequencing to reveal cell-state dependencies of genetic effects. Recent single-cell RNA-seq studies in in vitro models revealed changing genetic dependencies across different cellular transitions [5–7].

However, most existing analysis strategies for single-cell genetic studies have been based on computational methods originally developed for bulk-sequencing data [5, 6, 8], requiring the discretization of cellular states and thus potentially failing to detect more fine-grained differences in regulation. Computational strategies that allow for the unbiased identification of cell-state-specific effects are only beginning to emerge [9, 10] and currently rely on profiling a large number of genetically diverse individuals, which is particularly limiting for in vivo analyses and non-human model systems. The latter could be addressed by measuring allele-specific signals, i.e., quantifying molecular traits separately for each haplotype [9, 11–14], which in principle allows for identifying genetic effects even in a single individual. The combination of allele-specific quantifications coupled with the use of single-cell technologies could be a powerful strategy to dissect the functional impact of genetic variants both within and across multiple cell types contained in a complex tissue. Prior studies have quantified allele-specific properties at a single-cell level to characterize transcriptional bursting and stochasticity in gene expression [15, 16]. However, the analysis of allele-specific patterns to unravel allelic regulation at the single-cell level are only beginning to emerge [8, 17], and principled computational methods for this task are not established.

To address the aforementioned challenges, we developed a versatile computational model and analysis framework, scDALI (single-cell differential allelic imbalance). scDALI leverages allele-specific quantifications in single cells to identify and comprehensively test for different types of allelic effects, including homogeneous effects that are shared across all cell states or heterogeneous effects that are specific to some cell states. Intuitively, our model is similar in spirit to differential expression testing but aimed at identifying loci that exhibit heterogeneous allelic imbalance rather than variation in total expression. Critically, the model does not require the definition of cell states or clusters a priori and can cope with both discrete cellular states or continuous transitions. Additionally, scDALI enables the estimation of allelic imbalance from sparse sequencing data in individual cells, thereby facilitating the visualization and downstream interpretation of allelic regulation. scDALI is applicable to single-cell datasets obtained from different modalities and sequencing technologies.

We applied scDALI to study allele-specific variation in single-cell chromatin accessibility data (sciATAC-seq) in developing F1 embryos of *Drosophila melanogaster*, where we identified hundreds of regulatory regions with allelic imbalances in specific cell types or developmental stages. Among these effects, we identify putative enhancer regions with opposing allelic imbalance in different cell lineages, which are missed by bulk assay profiling. We then leveraged scDALI to fine-map the cell-type specificity of known expression quantitative trait loci (eQTL) in a population cohort of human induced pluripotent stem cells (iPSC), by assessing allelic regulation of single-cell transcriptomes. scDALI offered increased detection power compared to previous methods and uncovered how subtle differences in cell states can substantially affect allelic regulation. scDALI is therefore applicable to diverse species and data types, and leverages single-cell technologies to avoid cell sorting, thereby providing the means to discover and quantify the functional impact of cell state-specific genetic effects in a systematic and unbiased manner.

## Results and discussion

**Fig. 1:**
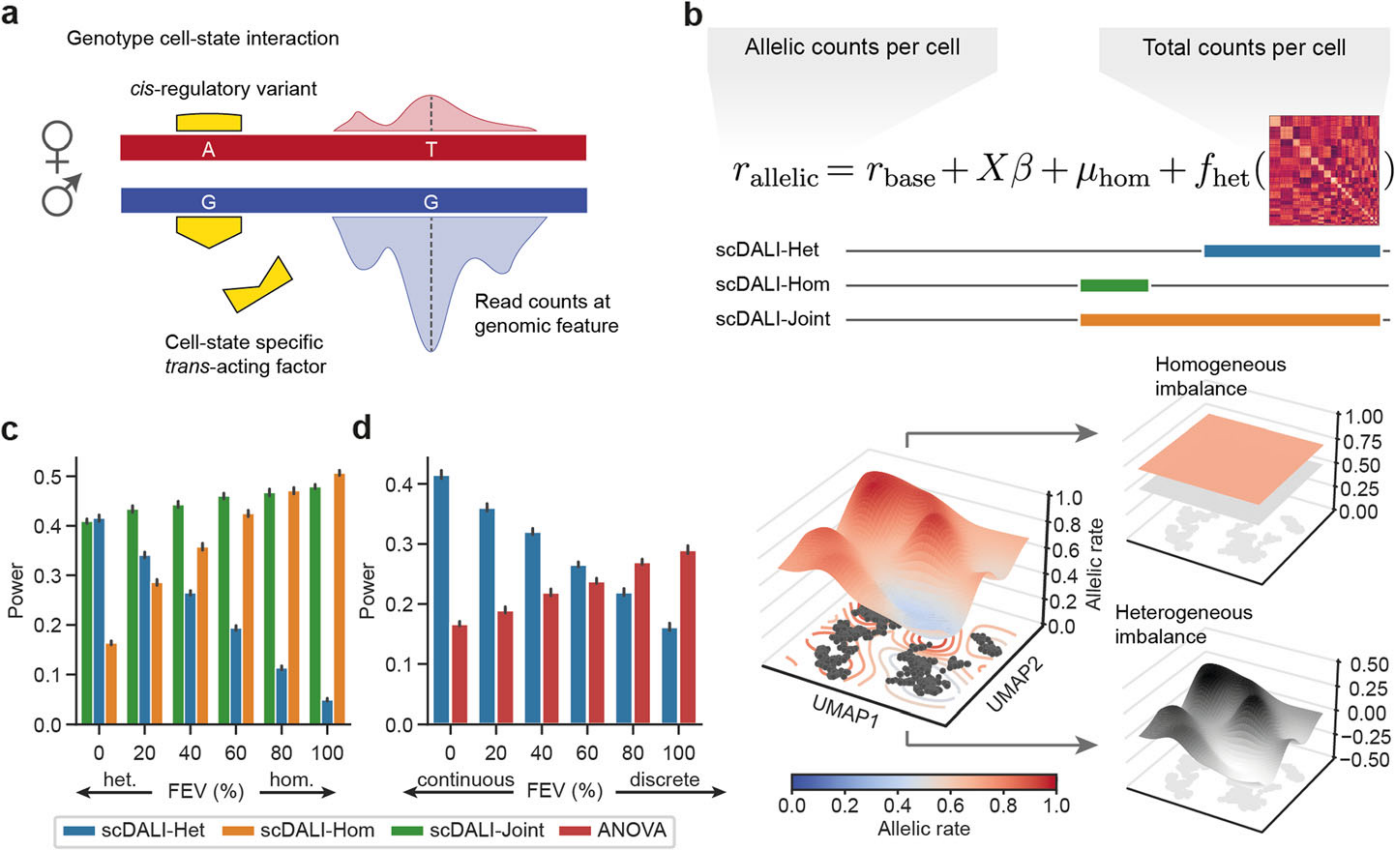
scDALI overview and model validation using simulated data. **a** Illustration of the causes and quantification of allelic imbalance. Heterozygous variants within quantified genomic features (T and G variant) are used to assign reads to either haplotype. A *cis*-regulatory genetic variant (A and G variant, yellow) impacts the efficacy of a *trans*-acting factor on the maternal allele, which results in allelic imbalance. If the *trans*-acting factor is cell-state specific, this effect will be heterogeneous across cell states, otherwise homogeneous. **b** Integration of total and allelic counts per cell. scDALI models allelic imbalance in single cells as the sum of a fixed expected rate (*r*_*base*_, e.g., 0.5 for autosomes), optional covariates, a global homogeneous component (*μ*_*hom*_), and a heterogeneous cell state-specific component (*f*_*het*_), which is characterized by a cell state kernel matrix. Three alternative score tests allow for assessing the evidence for global allelic imbalance (scDALI-Hom), heterogeneity across cell states (scDALI-Het), or to jointly test for either form of allelic imbalance (scDALI-Joint). **c**, **d** Assessment of power of alternative tests for allelic imbalance using simulated data. Allelic counts were simulated using alternative cell state kernels derived from real data (Additional file 1: Fig. S2, Methods). **c** Varying the simulated fraction of explained variance (FEV) of heterogeneous vs. homogeneous allelic imbalance. Whereas scDALI-Het and scDALI-Hom detect signals with simulated persistent or heterogeneous effects respectively, scDALI-Joint identifies either type of allelic imbalance. **d** Varying between simulating from a discrete and continuous cell state model. Shown are results from scDALI-Het and a baseline based on ANOVA to test for differences between discrete cell clusters (24 clusters; Methods)

scDALI enables the analysis of context-specific allelic regulation from single-cell sequencing data, either generated from outbred individuals or F1 crosses of inbred wild-isolates. Key to our approach is the integration of two independent signals that can be obtained from the same single-cell sequencing experiment: total counts and allele-specific quantifications. These signals can be derived from single-cell RNA-sequencing, single-cell ATAC-sequencing, and a range of other epigenetic assays. scDALI first uses total counts, quantified at individual features, to define a manifold of cell types and cell states, similar to established workflows for the inference of state clusters [18] or pseudo-temporal orderings [19]. Second, from the same dataset, allele-specific counts from matched cells are extracted, which allow for quantifying allelic imbalances and therefore genetic effects (Fig. 1a).

scDALI is a probabilistic model that can dissect dependencies between both of these signals, while aggregating evidence across cells to mitigate the sparsity of single-cell data. Briefly, our method can be cast as a generalized linear mixed model (GLMM) with a Beta-Binomial likelihood that accounts for count noise and residual overdispersion due to unmodeled variability in the data (Methods). This formulation extends the classical Beta-Binomial framework, which has previously been used for allele-specific analyses of bulk sequencing data [9, 12, 13, 20]. The model is also conceptually related to random effect models that have been proposed to study genotype-environment interactions in population-scale studies [10, 21]. scDALI captures both homogeneous deviation from a specified base allelic rate (e.g., 0.5 for autosomes in diploid organisms), as well as heterogeneity in allelic rates across cells using a kernel matrix that explains cell states, which is estimated from total counts [22, 23]. Our framework can capture a variety of cell state effects, including discrete cell clusters as well as continuous developmental trajectories. It is also possible to incorporate additional known covariates, such as batch or sample structure as fixed effects. Within the scDALI framework, we formulate computationally efficient score tests [24, 25] that allow us to identify sites that exhibit different types of allelic imbalance. In particular, scDALI implements tests specific to global *homogeneous* (pervasive) imbalance (scDALI-Hom), *heterogeneous* genetic effects that vary across cell types and cell states (scDALI-Het) or either of these effects (scDALI-Joint) (Fig. 1b). scDALI also allows for estimating the fraction of the total allele-specific variance that can be explained by cell state effects, and the model can be used to estimate and visualize allelic imbalances across cell states (Methods). Our framework is designed for the analysis of single-cell sequencing data from a small number of genetically distinct individuals, and hence the focus is not the discovery of novel quantitative trait loci but rather to leverage allelic imbalance to characterize the cell-state specificity of genetic factors.

### Model validation using simulated data

Initially, we validated our approach using simulated data, which was designed to mimic real count data as expected from a very heterogeneous sample (single-cell data from whole embryos), by adapting key parameters from empirical sciATAC-seq profiles from whole *Drosophila melanogaster* embryos (continuous and discretized cell states, overdispersion parameters; Additional file 1: Fig. S1a, 2, Methods). First, we assessed the calibration of all three scDALI tests by simulating from the corresponding null models, confirming uniformly distributed *P*-values (Additional file 1: Fig. S1b, c). Notably, a variant of scDALI-Het using a Binomial rather than Beta-Binomial observation model was not calibrated at overdispersion levels estimated from real data (Additional file 1: Fig. S1c). We also considered two alternative tests for modeling empirical allelic rates (maternal counts divided by total counts): a one-way ANOVA, testing for differences between discrete clusters and a multiple-degrees-of-freedom likelihood ratio test based on an ordinary least squares regression model (OLS, Methods). While the ANOVA model was calibrated, the OLS model led to inflated *P*-values when testing high-dimensional cell states relative to the sample size (Additional file 1: Fig. S1 d). Next, we simulated allelic counts from the scDALI model, varying the proportion of homogeneous versus heterogeneous allelic imbalance (Fig. 1c, Additional file 1: Fig. S2, 3a). As expected, scDALI-Joint identified effects of both classes, generalizing the individual tests scDALI-Het and scDALI-Hom. We then went on to simulate allelic counts either assuming continuous states, discrete cell state clusters derived from these states, or weighted combinations thereof (Fig. 1d, Additional file 1: Fig. S2, 3b, Methods). We compared scDALI to an ANOVA test based on the discretized cell state representation, finding that scDALI-Het offered substantial advantages in the presence of additional continuous variation, whereas ANOVA is most suitable to detect purely discrete effects. We also considered a range of additional settings, varying the levels of overdispersion and kernel variance (Additional file 1: Fig. S3), finding that scDALI was robust to a range of different parameters. scDALI is implemented as computationally efficient open-source software, scaling to the analysis of large datasets with up to tens of thousands of cells (Additional file 1: Fig. S4).

### scDALI identifies heterogeneous allelic imbalance in scATAC-seq from developing *Drosophila* embryos

**Fig. 2:**
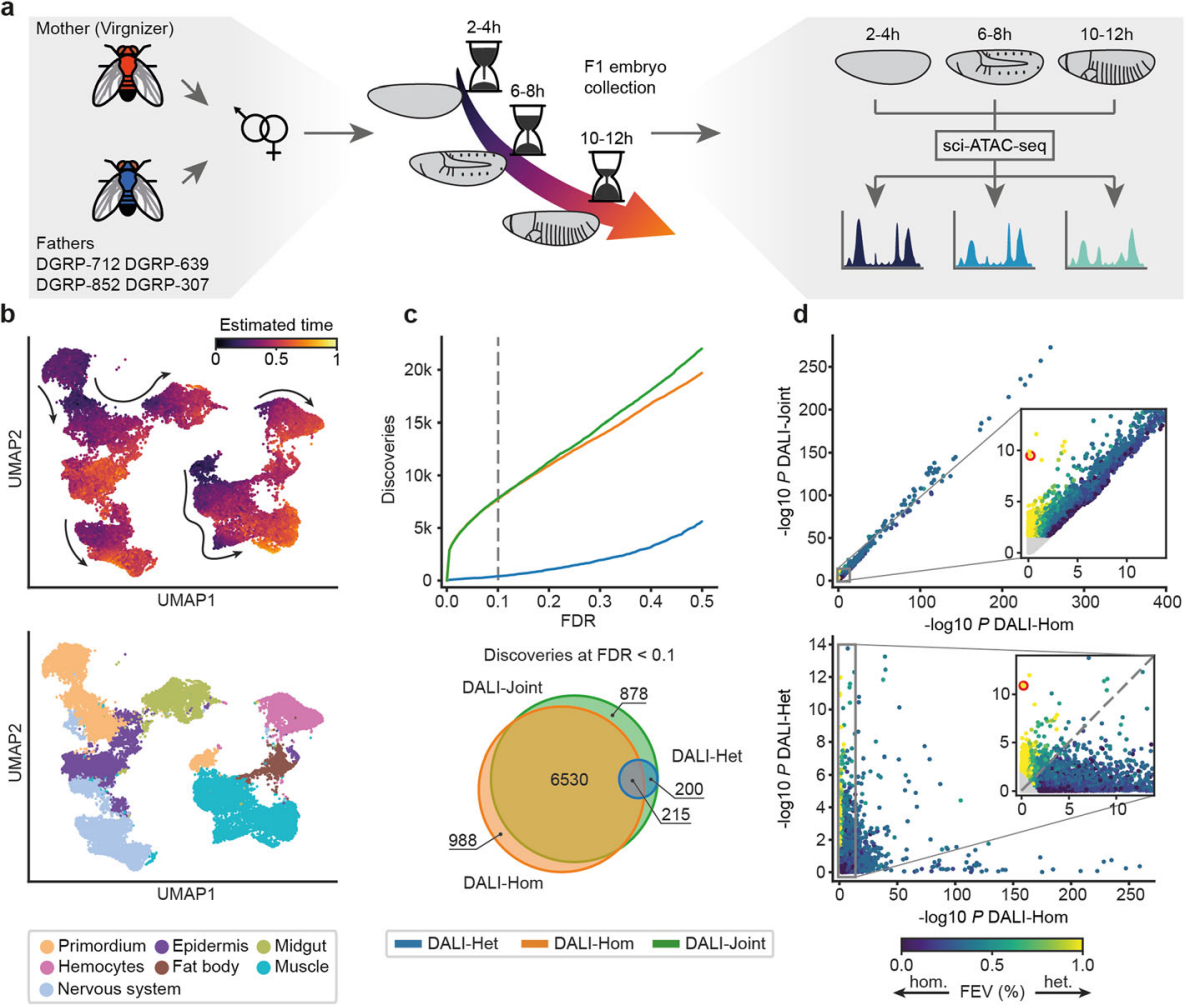
Application of scDALI to sciATAC-seq of *Drosophila* F1 embryos. **a** Experimental design. Chromatin accessibility was profiled in four F1 crosses at three distinct developmental stages (2–4, 6–8, and 10–12 h after egg laying), resulting in 12 sciATAC-seq libraries. **b** UMAP visualization of the full integrated dataset (34,053 cells from 12 sciATAC-libraries, excluding cell clusters with ambiguous annotations) based on the latent space of the Variational Autoencoder (VAE) (Methods). Top: Cells colors by the continuous temporal ordering as estimated from the VAE model. Bottom: Colored by the major lineage annotation. **c** Number of sites across crosses and time points with allelic imbalance identified by scDALI-Joint, scDALI-Hom, and scDALI-Het. Top: Number of discoveries as a function of the FDR threshold (Benjamini Hochberg adjusted). Bottom: Overlap between the sites identified by all three scDALI tests (FDR < 0.1). **d** Scatter plot of negative log *P*-values between scDALI-Joint and scDALI-Hom (top) and scDALI-Het versus scDALI-Hom (bottom) respectively. Color denotes the estimated fraction of allele-specific variance explained by cell-state-specific effects; non-significant peaks marked in grey (adjusted scDALI-Joint *P* > 0.1). Inset plots zoom in on peaks with pronounced cell state-specific imbalances. The red circle highlights the peak chr3R:20310056-20311056, a region showing prominent cell state-specific effects with no discernable global imbalance (c.f. Fig. 3a–c)

Having validated the model, we applied scDALI to open chromatin regions during embryonic development in F1 hybrid embryos of *Drosophila melanogaster.* We profiled single-cell chromatin accessibility by sciATAC-seq in F1 embryos obtained by mating the same mother to four genetically distinct fathers [20]. To ensure that we captured regulatory variation associated with major developmental events, we collected embryos from four F1 crosses at three key stages of embryonic development (Fig. 2a, 2–4 h, 6–8 h, and 10–12 h after egg laying), which correspond to stages when the majority of cells are multipotent, or are undergoing lineage commitment, and or tissue differentiation, respectively. Sequencing the resulting 12 sciATAC-seq libraries generated a dataset of 35,485 single cells (between 8000 and 10,000 cells per cross) that passed stringent quality metrics (Additional file 1: Fig. S5; Methods). Overall, our dataset features all the hallmarks of high-quality sciATAC-seq, including the appropriate nucleosomal banding pattern (Additional file 1: Fig. S5a), and a high concordance to previously identified peaks from a time-matched sciATAC-seq dataset in a reference strain [26] (Additional file 1: Fig. S6).

To infer a common cell state representation for all time points and crosses, we adapted a variational autoencoder (VAE) [27] that was previously developed for scRNA-seq data [28] to scATAC-seq. Briefly, a VAE is a neural network with a probabilistic bottleneck layer that learns the distribution of the data by compressing high-dimensional observations into a lower dimensional latent space. Our implementation (Additional file 1: Fig. S7a) incorporates a size-factor adjusted Bernoulli likelihood model tailored to the binary nature of scATAC-seq data. Furthermore, the model not only integrates measurements across datasets and batches but also allows to explicitly model information about different sampling times for developmental datasets (Additional file 1: Fig. S7d, e). This extension enables our model to infer continuous temporal ordering of cells by coupling the VAE objective function with a regression problem to predict sampling time from the latent cell state representation (Methods). We trained the model using the top 25,000 most accessible peaks across all crosses and time points. The VAE yielded a well-aligned latent space for all F1 crosses (Additional file 1: Fig. S7c) that captured progressive changes across developmental time (Fig. 2b, Additional file 1: Fig. S7d, e). We used the VAE latent space to define the cell state covariance for scDALI (see below). For annotating cell types, cells were clustered based on this lower-dimensional representation using the Leiden algorithm [29, 30] (28 clusters, Additional file 1: Fig. S7b), followed by an assignment of tissue identities based on the enrichment for enhancers with validated in vivo spatio-temporal activity in specific tissues during embryogenesis and genes with known tissue-specific expression [26] (Methods). Four smaller clusters with ambiguous annotations that likely correspond to barcode collisions were excluded from further analysis. This annotation process resolved seven cell populations that are representative of major embryonic lineages, including muscle, nervous system, and ectoderm (Fig. 2b).

Next, we quantified chromatin accessibility on an allele-specific level. We applied WASP [13] to avoid allelic mapping artifacts, filtering between 7 and 8% of mapped reads (Additional file 1: Fig. S8a, Methods). Allele-specific chromatin accessibility was quantified within 1 kb regions centered on ATAC peaks, requiring that each read overlapped at least one heterozygous variant. This resulted in a haplotype assignment for 20% of the reads (based on 5–6 variants per region on average, Additional file 1: Fig. S8b, c). After discarding peaks with low allelic coverage (mean count of reads that could be assigned to either allele < 0.1), we obtained between 8040 and 12,861 open-chromatin peaks per cross for further analysis resulting in a combined set of 39,530 peaks to be tested (Additional file 1: Fig. S9d, e).

We applied scDALI to test for homogeneous or heterogeneous allelic imbalance at each of the 39,530 peaks, jointly considering cells across all developmental stages for each cross (Fig. 2c, d, Additional file 1: Fig. S9; using the VAE latent space coordinates to define the cell state kernel; Methods). scDALI-Joint identified 7823 (~ 20%) ATAC peaks with evidence for allelic imbalance (FDR < 0.1, Benjamini-Hochberg adjusted). Notably, the majority of these peaks were also identified by scDALI-Hom (83%), indicating that homogeneous imbalance is prevalent. However, scDALI-Het identified 415 sites with evidence for cell state-specific allelic imbalance, 200 of which were missed by scDALI-Hom. This indicates that strong heterogeneity can preclude the identification of allelic imbalance by bulk sequencing or analysis strategies that assume exclusive homogenous effects. For instance, a peak (region chr3R:20310056-20311056) in cross F1-DGRP-307 was identified with high significance by scDALI-Joint (*P* = 1.93 × 10^−8^) and scDALI-Het (*P* = 5.45 × 10^−8^), but was globally consistent with a model that assumes no allelic imbalance (scDALI-Hom *P* = 0.81, Fig. 2d). We also assessed whether heterogeneous allelic imbalance at peaks identified by scDALI-Het could be explained by variation in total accessibility of the corresponding peak, finding no evidence for such a relationship for the vast majority of peaks (Additional file 1: Fig. S10 c, d). To evaluate to what extent scDALI is affected by the specific choice of cell-state representations, we also considered two alternative methods to define a cell state kernel, latent semantic indexing [26] (LSI), and cisTopic [31] (Methods). This comparison indicated that peaks with significant heterogenous imbalance were robustly identified across all three cell-state representations (Additional file 1: Fig. S10 a, b).

### Properties of regions with heterogeneous allelic imbalance

We applied scDALI to estimate allele-specific accessibility in individual cells for 415 peaks with significant heterogeneous allelic imbalance. We considered two alternative strategies for annotating cell-state-specific effects. First, we aggregated estimated allelic rates for each of the 7 annotated lineages and compared the rate distribution and mean allelic rates to identify lineages with pronounced differential allelic imbalance. Second, we estimated transcription factor (TF) activity scores for each cell based on the total accessibility of a curated set of 65 transcription factor motives [4] (using chromVAR [32]) and ranked TFs based on the correlation between their activity and estimated allelic rates (Methods). Notably, the latter approach avoids the definition of discrete cell clusters and thus can be used to identify specific regulatory programs associated with allelic imbalance.

**Fig. 3:**
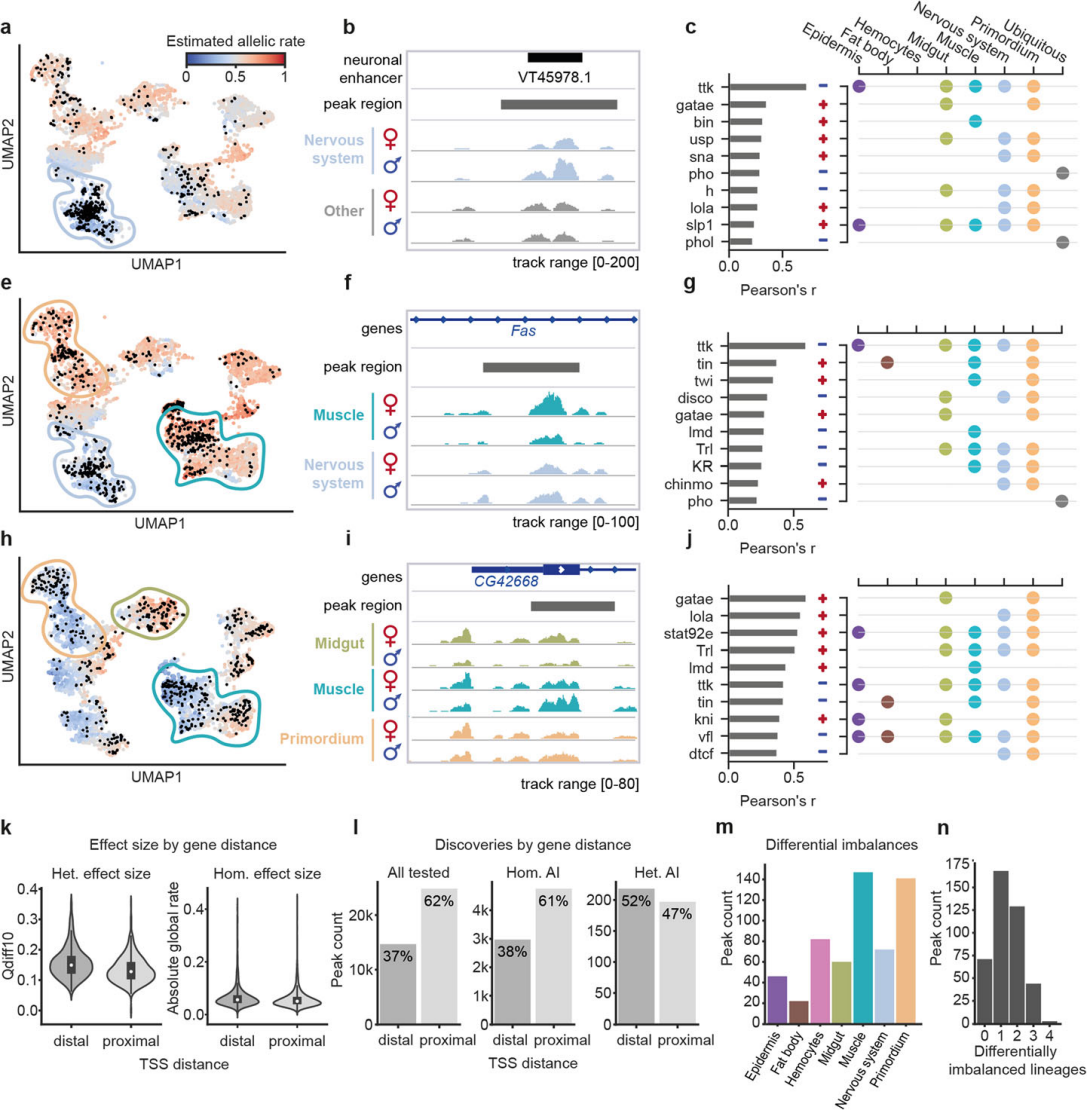
Examples and analysis of ATAC peaks with heterogeneous allelic imbalance. **a** UMAP visualization displaying cells colored by their predicted allelic rate (maternal accessibility relative to total accessibility) for region chr3R:22877489-22878489 in cross F1-DGRP-639. Black dots indicate cells with observed allelic rates (non-zero allelic total counts) that were used to fit the scDALI model. **b** Genome browser tracks for region chr3R:22877489-22878489 illustrating allele-specific aggregate accessibility for the nervous system and other populations. **c** Left: Correlation between estimated allelic rates and chromVAR transcription factor (TF) activity scores in individual cells. Shown are the ten strongest associations, with plus and minus signs indicating the direction of correlation. Right: Curated lineage annotation for each TF. **e**–**g** Example region chr2R:13675707-13676707 in cross F1-DGRP-639, revealing opposing effects in the nervous system and muscle lineage. **h**–**j** Example region chr3R:20310056-20311056 in F1-DGRP-307, revealing lineage-specific differences in allelic rates for the muscle, primordium, and midgut, as well as intra-lineage variation within the muscle population. **k** Violin plots of effect size estimates for heterogeneously (Qdiff10, i.e., 10% quantile difference as in **d**) and homogeneously imbalanced (absolute deviation from 0.5) peaks, considering distal and promoter-proximal regions separately. Distal peaks are associated with both larger absolute allelic imbalance and stronger heterogeneity (*P* = 5.6 × 10^−5^, *P* = 2.17 × 10^−26^, one-sided Mann-Whitney *U* test). **l** Total number of peaks tested and peaks with allelic imbalance identified using alternative tests (FDR < 0.1), stratified by the peak distance to the transcription start site (TSS) of the closest gene. Heterogeneously imbalanced peaks are markedly more common at distal regions. **m**, **n** By-lineage analysis of allelic imbalance using scDALI-Het for peaks with significant heterogeneous imbalances. **m** Distribution of the number of differentially imbalanced lineages per peak. **n** Distribution of the number of peaks with increasing numbers of differentially imbalanced lineages. The majority of peaks show imbalance in a single lineage

We find several cases in which allelic imbalance affects known lineage-specific regulatory elements. For example, region chr3R:22877489-22878489 (scDALI-Het *P* = 2.7 × 10^−5^) has been previously identified as a neuronal-specific DNase Hypersensitive Site (DHS) [33] and has been demonstrated to function as a nervous system enhancer in vivo (CAD4 database [26]). Accordingly, this region is identified as predominantly accessible in the nervous system (Fig. 3a). In addition, while cells from other lineages show no appreciable allelic imbalance, accessibility in the nervous system is strongly biased for the paternal allele (Fig. 3b, Additional file 1: Fig. S11b). Ordering cells by their estimated allelic rate and computing the difference between the top and bottom 10% quantiles (Qdiff10), we define a measure of the effect size of heterogeneous allele-specific imbalances, which captures the variation in allelic rates between the most extreme populations (Additional file 1: Fig. S11a). For this specific example, we obtain a Qdiff10 of 0.24 despite the overall mean allelic rate being close to 0.5 (Additional file 1: Fig. S11b). In accordance with the allelic imbalance identified by scDALI at this locus, the assessment of TFs associated with heterogeneity in allelic effects identified known nervous system regulators, such as Tramtrack (*ttk*) and Hairy (*h*) (Fig. 3c, Additional file 1: Fig. S11e).

Interestingly, we found a number of regulatory regions that show opposing allelic imbalances in different lineages. For example, region chr2R:13675707-13676707 has only a small maternal bias (estimated overall mean rate 0.61) when considering the global allelic rate but is identified as a site with pronounced allelic heterogeneity by scDALI (scDALI-Het *P* = 1.5 × 10^−8^, Fig. 3e). This region has previously been identified as a neuronal and muscle-specific DHS [33] and accordingly shows increased accessibility in the nervous system and muscle in our data. However, accessibility is biased for the maternal allele in the muscle and the paternal allele in the nervous system (Qdiff10 = 0.29, Fig. 3f, Additional file 1: Fig. S11c). This pattern of opposing allelic imbalance is also reflected in the correlation with the activity of TFs active in these tissues. For example, known muscle regulators, such as Twist (*twi*) and Tinman (*tin*) are correlated with the maternal allelic rate, while factors active in the nervous system, for example, Tramtrack (*ttk*), Disconnected *(disco*), and Kruppel (*Kr*), are correlated with the paternal rate (Fig. 3g, Additional file 1: Fig. S11f).

Another example is chr3R:20310056-20311056 (scDALI-Het *P* = 5.45 × 10^−8^), a region spanning an intron of the gene *CG42668*. The total accessibility of this region largely coincides with the known tissue-specific gene expression of *CG42668* in the cells of the midgut and visceral muscle. Our allele-specific analysis revealed differential allele-specific effects in both tissues, suggesting distinct regulatory programs orchestrating the tissue-specific activity of *CG42668* (Fig. 3h, i). Furthermore, muscle cells showed additional intra-lineage variation, resulting in a bi-modal distribution of allelic rates (Additional file 1: Fig. S11d). Despite the presence of strong inter- and intra-lineage variation (quantile difference 0.39), this effect is obscured in a bulk-level analysis (scDALI-Hom *P* = 0.81). The activity score of GATAe, a known midgut TF, is highly correlated (Pearson *r* > 0.5) with the maternal rate, while Zelda (*vfl*), which has a role in zygotic genome activation and early developmental patterning in the embryo primordium, with the paternal rate, consistent with the allelic bias observed in these cell populations (Fig. 3j, Additional file 1: Fig. S11g). The temporal intra-lineage variation within the muscle population is also reflected in the correlation with the activity of known early and late muscle TFs. Twist (*twi*) and Tinman (*tin*) are active in the early muscle primordium (mesoderm) where they direct the specification of the muscle lineages, and concordantly their activity scores are correlated with the paternal allelic rate observed in the early muscle cells. TF Lameduck (*lmd*) is instead correlated with the maternal rate, as it is required during later stages of muscle formation for the proper specification of the somatic and visceral muscle (Fig. 3j, Additional file 1: Fig. S11g).

More globally, allele-specific effects are stronger at distal regulatory elements (potential enhancers) compared to promoter-proximal regions, both for peaks with heterogeneous (one-sided Mann-Whitney *U* test, *P* = 5.6 × 10^−5^) as well as homogeneous (one-sided Mann-Whitney *U* test, *P* = 2.17 × 10^−26^) imbalance (Fig. 3k). Furthermore, imbalances are significantly more common at distal versus proximal regions (Fig. 3l), similar to what has been observed in bulk ATAC-seq data at time-matched developmental stages [20]. These differences between distal and proximal sites are less pronounced when considering discoveries from scDALI-Hom (two-sided Binomial test *P* = 0.02), with about 61% of significant regions being found at proximal regions compared to 62% of all tested peaks. Interestingly, however, we find this effect to be markedly more prominent for heterogeneously imbalanced regions (two-sided Binomial test *P* = 2.15 × 10^−10^), with only 47% of peaks discovered by scDALI-Het being located near gene promoters.

To further characterize heterogeneous imbalances, we used scDALI to assess differential lineage effects, testing for differences in mean allelic rates between each lineage and all remaining cells (Methods). Briefly, this test can be formulated under the scDALI-Het framework, replacing the continuous cell state kernel with a block-diagonal matrix to indicate lineage membership. Unsurprisingly, the frequency of significant imbalances by lineage (FDR < 0.1) largely resembled the overall read count distribution, which influences the detection power for allelic imbalance (Fig. 3m, Additional file 1: Fig. S12a). For the majority of peaks, allele-specific variation was attributable to one or two differentially imbalanced lineages (72%); however, 11% of peaks showed differences between three of four lineages (Fig. 3n). Interestingly, for 17% of scDALI-Het discoveries, allele-specific effects do not differentiate any single lineage, indicating the presence of significant intra-lineage variation, for example due to variation in developmental time.

### Identification of sites with heterogeneous allelic imbalance linked to developmental time

**Fig. 4:**
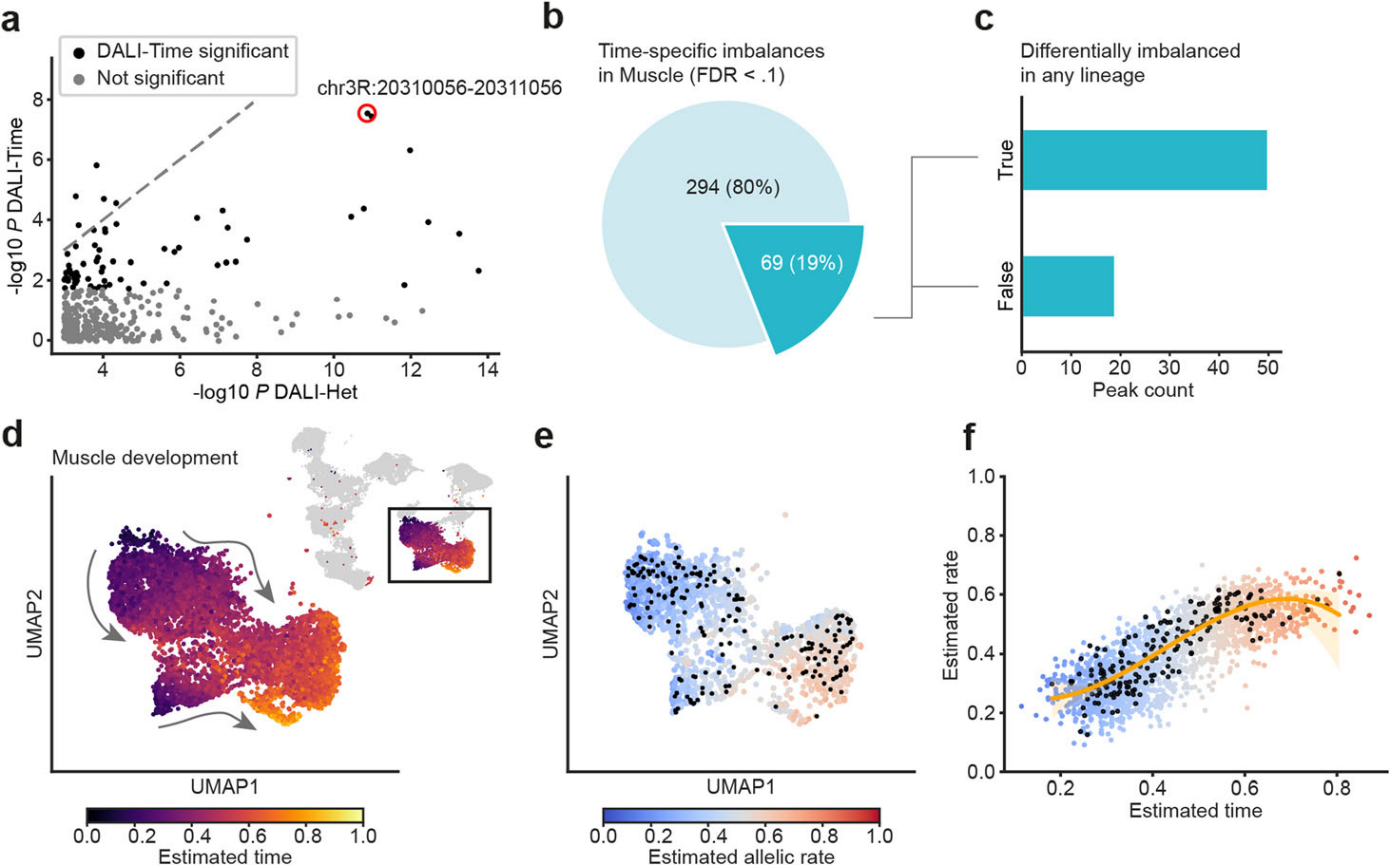
scDALI-Het identifies time-specific intra-lineage variation. **a** Scatter plot of negative log *P*-values for scDALI-Het versus a scDALI test for time-specific variation in the muscle population. Red circle highlights region chr2R:13675707-13676707. **b** Of 363 peaks identified by scDALI-Het that are accessible in the muscle, 19% showed significant temporal effects (FDR < 0.1). **c** The majority of peaks with a time-specific effect in the muscle did not show significant differential allelic imbalance between lineages. **d** Temporal order for the muscle lineage estimated by the variational autoencoder model. **e**, **f** Estimated allelic rates across time for region chr2R:13675707-13676707. Black dots denote cells with observed allele-specific counts in this region

Developmental time is a major driver of variation in our dataset and therefore a promising predictor of allele-specific changes within lineages. We applied scDALI to test for time-specific allelic imbalances within muscle, the lineage with the largest number of cells, using the pseudo-temporal ordering estimated by the VAE model as a cell state representation (Fig. 4d). Leveraging the scDALI framework, we design a kernel capturing both linear and nonlinear (polynomial) temporal dependencies (Methods). Out of 363 peaks with significant heterogeneous allelic imbalance that are accessible in muscle (mean total allelic count within lineage < 0.1), scDALI identified 69 (19%) peaks with significant time-specific effects (FDR < 0.1; Fig. 4a, b). Notably, 27% of these peaks with time-specific allelic imbalance did not show any lineage-specific effects (Fig. 4c). As an example, region chr2R:13675707-13676707 discussed above (Fig. 3f) does indeed exhibit strong time-specific imbalances (Fig. 4e, Fig. 4f), consistent with the observed intra-lineage variation specifically in muscle cells.

### Application of scDALI to identify cell-type-specific effects of eQTL

To demonstrate that scDALI is also applicable to single-cell RNA-seq, we considered a recently published multi-donor single-cell RNA-seq dataset of human induced pluripotent stem cells (iPSCs) differentiating towards definitive endoderm [5]. Samples were profiled using a full-length sequencing protocol (Smart-seq2, [34]), allowing for the quantification of gene expression in haplotype-resolved manner and thus providing the basis for an analysis using scDALI. Briefly, this study spans 34,254 cells (after basic filtering, Methods) from 125 donors at four time points during cell differentiation. We considered 3966 eQTL (SNP-gene pairs) that were identified in the primary analysis and applied scDALI-Het to assess the evidence for heterogeneous allelic gene expression. Briefly, for each of these eQTL, we aggregated allele-specific counts across all cells from donors with a heterozygous query eQTL variant relative to this variant (using haplotype phasing, c.f. Methods and [5]). We then used the first 20 principal components from total expression counts to construct a cell state kernel for the scDALI analysis (Methods).

**Fig. 5:**
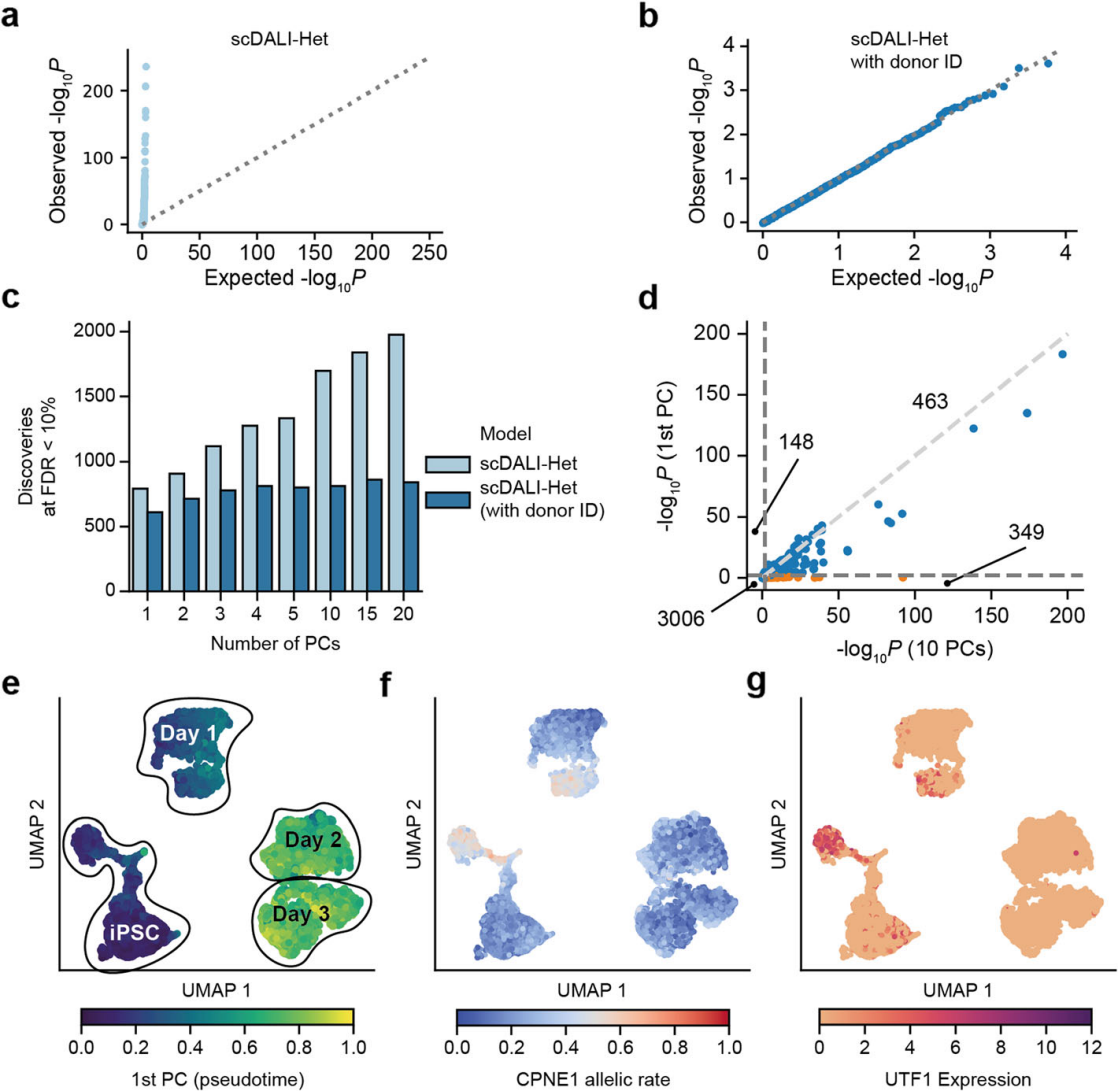
scDALI-Het applied to scRNA-seq of differentiating iPSCs reveals cell-state specificity of eQTL. **a**, **b** Q-Q plot of scDALI-Het *P*-values when permuting the cell-state coordinates of cells from the same donor. A model that does not account for the donor identity yields inflated *p*-values (**a**), whereas scDALI-Het with donor identities as fixed effects yields calibrated results (**b**). **c** Number of discoveries for varying numbers of principal components (PCs) used to define the cell state kernel. If donor identity is not accounted for, using a larger number of PCs for the cell-state definition leads to an increasing number of discoveries. **d** Scatter plot of negative log P-values, comparing a model using only the leading PC versus scDALI-Het with 10 PCs. Orange dots are discoveries that are exclusively identified by the general cell-state test (10 PCs). Indicated are the number of significant discoveries in each quadrant (10% FDR). **e** UMAP visualization of collection- and pseudotime. **f** Estimated allelic rates for the eGene-QTL pair (CPNE1, chr20:34344225 T/A). **g** Expression of UTF1, a previously identified marker for neuronal differentiation success

While allelic rates are generally less susceptible to confounding variables such as batch effects, donor-specific read mapping biases as well as differences in the representation of cell types and cell states can lead to spurious signals of heterogeneous allelic variation. Indeed, we confirmed the need to account for the donor identities (donorID) in this analysis to retain calibrated test statistics (10 PCs, Fig. 5a, b; assessed using permuted cell coordinates; Methods).

We assessed the number of eQTL with heterogeneous imbalance discovered by scDALI-Het when varying the number of principal components used to construct the cell-state kernel, finding that more complex kernels yielded a larger number of discoveries, which however saturated for five or more components (Fig. 5c). For example, a model using the first PC to define a cell state kernel (which primarily captures differentiation, Additional file 1: Fig. S13) identified 611 eQTL with heterogeneous allelic imbalance compared to 812 eQTL when using 10 components (Fig. 5d, FDR < 0.1). This indicates that although variation in gene expression in this data is predominantly explained by the differentiation state (Fig. 5e), the remaining sources of variation drive a substantial fraction of distinct genetic regulation. One example of such an effect is an eQTL with heterogeneous ASE for CPNE1 (*P* = 3 × 10^−9^, scDALI-Het). CPNE1 has been shown to play a role in neuronal progenitor cell differentiation [35]. Intriguingly, the pattern of allelic imbalance is confined to a distinct subpopulation of iPS cells, which is marked by expression of UTF1. Notably, this UTF1-positive iPS subpopulation has recently been associated with differentiation efficiency towards a midbrain neural fate [6] (Fig. 5g).

## Conclusion

The majority of disease associated variants impact non-coding regions, disrupting the function of regulatory elements such as enhancers and promoters. As enhancers regulate when and where genes are expressed, genetic variation within enhancers naturally has cell type-specific effects. However, capturing and understanding these genetic effects is an enormous challenge. Resolving these effects to specific cell types using classical quantitative trait loci (QTL) mapping would require FACS sorting different cell types from a heterogeneous tissue across a large panel of individuals, a huge task that is often impossible as specific markers for cell isolation are not available for many cell types and transitions.

To address this, we developed scDALI, a computational framework to characterize the cell-type specificity of genetic effects from single-cell sequencing data in an unbiased fashion. Our model provides a principled strategy for exploiting two independent signals that can be obtained from the same sequencing experiment, whether that is gene expression or epigenetic data: (1) total counts, which we use to derive cell types and states, and (2) allele-specific quantifications of genetic effects within genomic features such as genes or ATAC peaks of accessibility. Combining these two measurements allowed us to test for both pervasive, homogeneous imbalance and cell-state-specific heterogeneous effects, without the need to define cell types or cell states a priori.

We applied scDALI to newly generated scATAC-seq profiles from an F1 cross design, assaying dynamic and discrete changes in allele-specific chromatin accessibility of developing *Drosophila melanogaster* embryos, a naturally very heterogeneous sample. Our model discovers thousands of imbalanced regions, hundreds of which show distinct cell state-specific effects. About half of the regulatory regions with allelic imbalance in specific cell types are not detectable in a pseudo-bulk analysis, as opposing effects cancel out across the cell state space. Although the total number of discoveries with heterogeneous effects is relatively modest, we expect this to increase dramatically as the number of profiled cells increases. Even with the numbers profiled here, our analysis identified genetic effects at a number of characterized tissue-specific developmental enhancers. scDALI estimates allele-specific effects in individual cells, which allows dissecting this heterogeneity at different resolutions. We have shown how this map can be used to identify the underlying regulatory programs by associating differential allelic imbalance with pathway or transcription factor activity scores. Alternatively, it is possible to aggregate allelic rates at the level of known (discrete) clusters, thereby assessing the distribution of estimated allelic activity both between and within lineages or cell types. We find that developmental time is an important contributor to intra-lineage variation of allelic imbalance, pinpointing developmental stage-specific enhancers. Furthermore, our analysis revealed that allele-specific effects are significantly stronger and more common at distal elements (putative enhancers) compared to promoter-proximal regions. Notably, these differences are markedly more pronounced among peaks with heterogeneous (tissue-specific) imbalances compared to homogeneous effects, confirming and extending previous results on bulk-sequencing data [20]. We then applied scDALI to a published scRNA-seq dataset from 125 human iPS cell lines and demonstrated how our model can be used to discover context-specific genetic effects of known eQTL and characterize the associated cellular subpopulations.

While our approach uncovers many novel putative enhancers, it also has its limitations. The focus of this work lies on the characterization of cell-state-specific effects for known quantitative trait loci and the mapping of genetic effects from few available individuals or even a single sample. In particular, we do not test for interactions between cell states and the presence of genetic variants, which prevents our model from discovering potential causal loci associated with cell-state-specific allelic imbalance. While in principle, it is possible to combine allelic analyses with genotype data to identify causal variants [9, 11–13], this requires larger numbers of unique genotypes. Furthermore, even for population-scale studies using single-cell sequencing, the primary interest is the characterization of known loci and not discovery of novel effects. These considerations are motivated by differences in power to detect eQTL in bulk versus single-cell data [36] and the sample sizes that can currently be profiled using single-cell readouts. The required multi-individual single-cell sequencing studies are only beginning to emerge and scDALI could be extended to leverage such variation.

Understanding to what degree allele-specific effects replicate at different molecular layers remains another important direction of future research. In this study, we have demonstrated that scDALI can be flexibly applied to both single-cell RNA-seq and ATAC-seq data. However, new multi-omics methods can obtain both DNA accessibility and RNA measurements from the same single cell [37]. The integration of these different dimensions of allelic imbalance across both modalities will be an important area for future work that may help to relate the functional impact of genetic variation in enhancers to their target gene’s expression.

## Methods

### scDALI model

scDALI extends the frequently used Beta-Binomial observation model for allele-specific read counts in bulk-sequencing data [9, 12, 13, 20], by accounting for cell-state-specific effects. For a given genomic region of interest and cells *i* = 1, …, *n* let *a*_*i*_ be the number of reads mapping to the the maternal haplotype and *d*_*i*_ be the total number of reads. Furthermore, let *K* denote a *n* × *n* cell-state kernel matrix, capturing cell-to-cell covariances across the cell state space. Throughout our analyses, we use *K* = *EE*^*T*^ where *E* is a low-dimensional representation based on total read counts. For example, *E* can be obtained by reducing the dimensionality of the total counts matrix using principal component analysis (PCA) or a variational autoencoder model (see below) or by constructing a (one-hot-encoded) cell clustering. scDALI captures cell-state-specific allelic variation on a logit scale using a latent *n*-dimensional Gaussian variable:

1 $$ u\sim N\left(1\cdotp \alpha + X\beta, {\sigma}^2K\right) $$

Here, the scalar *α* denotes global or *homogeneous* allelic imbalance, affecting all cells equally and independent of the cell state, while *σ*^2^ modulates the strength of cell-state-specific or *heterogeneous* effects. To couple *u* to the mean of a Beta-Binomial observation model for allelic read counts, scDALI then uses a logit link function *g*(*x*) =  *log* (*x*/(1 − *x*)):

2 $$ {\mu}_i={g}^{-1}\left({u}_i\right) $$

3 $$ {a}_i\mid {\mu}_i,{d}_i\sim BetaBinom\left({\theta}^{-1}{\mu}_i,{\theta}^{-1}\left(1-{\mu}_i\right)\right) $$

The parameter *θ* captures residual, extra-binomial variance (overdispersion) due to unmodeled technical and biological sources of variation. Note that by using a linear kernel function *K* = *EE*^*T*^, we effectively cast our model as a generalized linear mixed model [38] (GLMM). However, the model can in principle be extended to the non-linear case using common kernel functions from the Gaussian process literature [39] or using non-linear transformations of individual cell-state dimensions.

To systematically assess homogeneous or heterogeneous allelic imbalance, scDALI implements three score tests:

- scDALI-Het *H*_0_^*Het*^ : *σ*^2^ = 0 *vs*. *H*_1_^*Het*^ : *σ*^2^ > 0 (heterogeneous imbalance)
- scDALI-Hom *H*_0_^*Hom*^ : *α* = 0 *vs*. *H*_1_^*Hom*^ : *α* ≠ 0 (homogeneous imbalance)
- scDALI-Joint *H*_0_^*Joint*^ : *α* = 0, *σ*^2^ = 0 *vs*. *H*_1_^*Joint*^ : *α* ≠ 0 *or σ*^2^ > 0 (general imbalance)

By leveraging a score-based framework, scDALI avoids fitting the full GLMM model under the alternative hypotheses when evaluating the test statistics, which is computationally expensive for large data sets. The associated null models can be fitted efficiently using the Fisher scoring/Newton-Raphson. For a full derivation of the scDALI score tests, see Additional file 2: Supplementary Methods.

### Allelic rate interpolation and downstream analysis

Once a set of genomic regions with significant heterogeneous allelic imbalance has been identified, scDALI can be used to estimate the landscape of allelic imbalance across the cell state space for the purpose of visualization and downstream analysis. For computational reasons, we approximate the scDALI model described above, replacing the Beta-Binomial observation model (3) with a Gaussian likelihood model for empirical rates *r*_*i*_ = *a*_*i*_/*d*_*i*_. Both model parameters (equation (1)) and posterior approximations for allelic rates can be fitted efficiently using sparse variational inference [40, 41].

While the estimate for *α* provides a measure of pervasive, homogeneous allelic imbalance, we can use the posterior mean of the latent variable *u* as an estimate for cell-specific allelic rates. In particular, we define a measure of effect size or statistical dispersion for heterogeneous effects, *Qdiff10*, as the difference between the 90% and 10% quantiles of the estimated posterior mean for *u*.

### Guidelines for the cell state definition

An appropriate cell-state definition depends on the data as well as the research question. If differences in allelic rates between discrete cell types are of primary interest, using a one-hot encoding of the cell type clusters will maximize detection power for these effects. However, such a representation will ignore continuous, e.g., intra-cell type variation and results will depend on whether or not the cell clustering represents a biologically meaningful discretization. In some cases, a continuous representation is a natural choice, e.g., when studying differentiation or developmental time courses. As a general-purpose approach, we propose to use a lower-dimensional embedding of the total counts matrix for the detection of both continuous and discrete effects. Similar to the standard analysis of single-cell sequencing data, the choice of a particular dimensionality reduction method should be informed by a variety of factors (dataset size, need for interpretability, specific characteristics of the data modality, etc.).

### Guidelines for the control of confounding effects

In many cases, both alleles are thought to be affected similarly by technical confounders and batch effects, and consequently, these effects will cancel out when quantifying allelic rates. However, all factors that may affect *rates* rather than allelic counts need to be accounted for, e.g., possible individual-specific reference mapping biases in a population-scale analysis (see section Analysis of allelic imbalance in population-scale iPSC data). Nevertheless, we advise to adjust for common technical confounders (batch effects, size factors) when constructing the cell-state representation, which is typically based on total read counts.

### Cell state variational autoencoder

To infer a lower-dimensional embedding from chromatin accessibility profiles of temporally resolved scATAC-seq data, we implement a variational autoencoder model [27] (VAE). Variations of VAE models have been widely applied to model single-cell transcriptome measurements [28, 42, 43] and more recently been extended to model chromatin accessibility data [44]. Our model is most closely related to scVI [28], a VAE capable of integrating scRNA-seq data across different individuals or batches while accounting for library-size variation. However, our model differs from scVI in two notable aspects. First, we use a likelihood model tailored to the near-binary nature of single-cell ATAC-seq data. Second, we integrate sampling times for developmental datasets to estimate a continuous pseudo-temporal ordering from few available time points. The model can be decomposed into three sub-modules (Additional file 1: Fig. S1a): the *decoder network*, representing the generative process for observed accessibility profiles, the *temporal classifier*, and the *encoder network* for inferring the posterior distribution over latent variables.

Let *x*_*i*_ ∈ {0, 1}^*m*^ be the binarized accessibility vector for *m* peaks in cell *i* and *c*_*i*_ be the batch / individual identity. The probabilistic generative model underlying the decoder module is as follows:

$$ {z}_i\sim N\left(0,I\right) $$

$$ {l}_i\mid {c}_i\sim LogNormal\left({\mu}_l\left({c}_i\right),{\sigma_l}^2\left({c}_i\right)\right) $$

$$ {\rho}_i={f}_{\rho}\left({z}_i,{c}_i\right) $$

$$ {x}_{ij}\mid {\rho}_{ij},{l}_i\sim Bernoulli\left(1-{\left(1-{\rho}_{ij}\right)}^{l_i}\right) $$

Here, *z* are the latent, low-dimensional cell state representations, and *l*_*i*_ is a cell-specific size-factor variable capturing variation in sequencing depth [28]. The prior parameters *μ*_*i*_(*c*_*i*_) and *σ*_*l*_^2^(*c*_*i*_) are chosen to be maximum-likelihood estimates based on the total number of reads per cell in each cross. Cell states along with observed batch ids are mapped to *m*-dimensional peak activities *ρ*_*i*_ ∈ [0, 1]^*m*^, ∑_*j*_*ρ*_*ij*_, representing the relative “openness” of each peak in cell *i*. The mapping is realized by a neural network *f*_*ρ*_ with trainable parameters. By providing both *f*_*ρ*_ and the encoder network (see section below) with *c*_*i*_, the model is encouraged to disentangle batch-specific effects and cell state representations [28, 45] (Additional file 1: Fig. S1c). The full distribution over observed accessibility profiles is obtained by applying the scaling factor to the peak activities. If *l*_*i*_ were the true (discrete) number of reads per cell, $1-{\left(1-{\rho}_{ij}\right)}^{l_i}$ would correspond to the probability of observing at least one read in peak *j*. However, to simplify the inference process, we place a continuous log-normal prior on *l*_*i*_.

For the *Drosophila melanogaster* data considered in this paper, coarse temporal information in the form of embryo collection windows is available (Additional file 1: Fig. S1d). We integrate these time stamps with the observed ATAC-seq data to inform the latent-space inference, correct time measurement errors, and learn a continuous ordering of cells from few available labels. Assume the time label *y*_*i*_ for cell *i* takes on one of *t* ordered values. If *t* is small, it is difficult to accurately estimate the temporal scale at which cell state changes take place. Instead, we model the relative order of cells as a function of the cell state *z*_*i*_, using an ordinal likelihood model. Formally, we assume *y*_*i*_ ∈ {1, 2, …, *t*} and define [46]

$$ p\left({y}_i\ |\ {z}_i\right)=\varPhi \left({w}_{y_i}-{f}_y\left({z}_i\right)\right)-\varPhi \left({w}_{y_i-1}-{f}_y\left({z}_i\right)\right) $$

where *Φ* denotes the cumulative distribution function of the standard normal distribution, *w*_0_ =  − ∞, *w*_*t*_ = ∞ and *w*_1_, …, *w*_*T* − 1_ are trainable parameters such that *w*_*i*_ < *w*_*i* + 1_. The function *f*_*y*_ maps cell states to pseudo-temporal values along the real axis and is chosen to be a simple linear model. Intuitively, *p*(*y*_*i*_ | *z*_*i*_) corresponds to the probability of sampling a value from the interval $\left({w}_{y_i-1},{w}_{y_i}\right)$ under a normal distribution with mean *f*_*y*_(*z*_*i*_) and unit variance. By allowing for Gaussian noise around the latent time *f*_*y*_(*z*_*i*_), we can account for measurement errors in the labeling process. Note that the *w*_*i*_ form a contiguous segmentation of the real line which enforces ordinal constraints. Guided by both observed time stamps and cell state proximities, the model infers a high-resolution pseudo-temporal trajectory *f*_*y*_, allowing us to order cells according to their developmental progression.

We optimize all parameters jointly using amortized variational inference [27], incentivizing the model to learn a cell state representation that is informed by and supports the observed time labels (Fig. 2b, Additional file 1: Fig. S1e). For a full description of the mathematical details of the variational approximation and practical implementation details, we refer the reader to the Additional file 2: Supplementary Methods.

### Generation and sequencing of *Drosophila melanogaster* F1 embryos

We generated *Drosophila melanogaster* F1 hybrids by crossing females from a common maternal virginizer line with males from four different inbred lines from the *Drosophila melanogaster* genetic reference panel [20, 47] (DGRP). Embryos were collected in 2 h windows (2–4 h, 6–8 h, and 10–12 h after egg laying) as previously described [20].

Hyperactive Tn5 transposase was purified by the EMBL Protein Expression and Purification facility as previously described [48] and stored at − 20 °C in storage buffer (25 mM Tris pH 7.5, 800 mM NaCl, 0.1 mM EDTA, 1 mM DTT, 50% glycerol) until use. Uniquely indexed oligonucleotides from Cusanovich et al. [26] were annealed to common pMENTs oligos 95 °C 5 min, cooling to 65 °C (0.1 °C/s), 65 °C 5 min, cooling to 4 °C (0.1 °C/s)) to generate indexed transposons that were then loaded onto purified Tn5 by incubation at 23 °C with constant shaking at 350 rpm for 30 min. The loaded Tn5 transposomes were diluted 1:10 (final 0.02 mg/ml) in nuclease-free water and used immediately for tagmentation.

Embryo dissociation and nuclear isolation were performed as described previously [26]. Nuclei were flash frozen in liquid nitrogen and stored at − 80 °C until use. Generation of sci-ATAC-seq libraries was performed largely as previously described [26], with minor modifications. The tagmentation reaction was performed by adding 2 μL of each of the 96 custom and uniquely indexed Tn5 transposomes and by incubating at 55 °C for 1 h. After reverse-crosslinking, 5 μL of forward and reverse indexed primers (from Cusanovich et al. [26]), 7.5 μL KAPA HiFi DNA Polymerase ReadyMix (Roche) and 0.25 μL Bst3.0 (NEB) were added to each well. Tagmented DNA was then PCR amplified with the following cycling conditions: 72 °C 5 min, 98 °C 30 s; 98 °C 10 s, 63 °C 30 s, 19–22 cycles; 72 °C 1 min, hold at 10 °C. The optimal number of cycles for each library was determined beforehand by monitoring amplification on a qPCR machine for a set of test wells. Libraries were sequenced on an Illumina NextSeq 500 sequencer High Capacity 150 PE kit as previously described [26].

### Processing of raw sci-ATAC sequencing data

Raw sequencing data was processed based on the pipeline (https://github.com/shendurelab/fly-atac/) developed by Cusanovich et al. [26]. BCL files were converted to fastq files using bcl2fastq v.2.16 (Illumina). To correct for sequencing or PCR amplification errors, read barcodes were matched against all possible barcodes. In case of an approximate match (Levenshtein distance < 3 and distance to next best match > 2), the corresponding barcodes were fixed to their presumptive match; all other barcodes were classified as ambiguous or unknown. Barcode correction was followed by adapter trimming [49] and read alignment to the dm6 reference genome using bowtie [50] (with options -X 2000 -3 1). After the removal of PCR duplicates, we classified barcodes corresponding to genuine cells from the background by fitting a two-component Gaussian mixture model to the log-transformed read counts per barcode. A cutoff for cell barcodes was determined by requiring that the posterior probability of belonging to the higher read-depth mixing component was greater than 0.95 (Additional file 1: Fig. S6b, c).

Chromatin accessibility was quantified in a set of 53,133 peaks of accessibility previously identified from a time-matched sci-ATAC-seq dataset [26] and lifted to the dm6 reference genome (https://github.com/FlyBase/bulkfile-scripts). We compared the Pearson correlation between pseudo-bulk aggregates for each collection window both within our dataset as well as between our data and the published reference (Additional file 1: Fig. S7). Next, we restricted our analysis to autosomes in order to remove sex-specific biases [26]. For each cross and collection window, we determined the 10% and 99% quantiles of the cell-count distribution and only kept cells whose counts were within those limits, resulting in 35,485 cells in total. Finally, we selected the 25,000 top most accessible peaks for further analysis. We trained the cell state variational autoencoder on the whole dataset for 30 epochs. Additionally, we generated alternative representations for data from cross F1-DGRP-712 using LSI [26] (leading components 2 to 20; the first component was excluded due to correlation with total counts per cell) and cisTopic [31] (50 topics). These embeddings were only used to assess the robustness of the scDALI workflow to different cell state representations (Additional file 1: Fig. S10).

We used the Scanpy implementation of the Leiden algorithm [29, 30] with a resolution of 1.2 and identified 28 cell clusters in the joint VAE latent space. For each cluster, we computed differentially accessible peaks using logistic regression by predicting cluster labels from the relative peak activity profiles obtained from the VAE model [30, 51]. We then performed an enrichment analysis for known tissue specific enhancer elements (CAD4 database [26] and genes (tissue-specific expression of the nearest gene based on in situ hybridization data from the Berkeley *Drosophila* Genome Project (http://insitu.fruitfly.org/cgi-bin/ex/insitu.pl) and FlyBase gene expression annotations (http://flybase.org/) using a Fisher’s exact test. Based on these enrichments, each cluster was assigned to one of seven major lineages (Fig. 2b). Four clusters with a total of 1432 cells could not be annotated unambiguously and were removed from further analysis (Fig. 1b), resulting in a final set of 35,485 cells (Additional file 1: Fig. S1d).

### Processing of allele-specific sci-ATAC counts

We used existing genotyping information [20] for the parental strains to create cross-specific VCF files, filtering for genetic variants that were heterozygous in the F1 generation. To eliminate reference mapping biases, we applied the WASP pipeline [13] (https://github.com/bmvdgeijn/WASP/tree/master/mapping) and created filtered BAM files, removing between 7 and 8% of mapped reads (Additional file 1: Fig. S8a) from the original alignment. For each of the 35,485 cells, we then quantified allele-specific accessibility by adapting the original WASP code for count generation to single-cell sequencing (https://github.com/tohein/scai_utils). As features, we chose 1 kb windows centered on each of the 53,133 peaks to mitigate the inherent sparsity of the data. Reads aligned within these windows were assigned to either allele, requiring that each read overlapped at least one heterozygous single-nucleotide variant. If a read overlapped multiple variants, one was chosen at random to determine the allele of origin. As it can be challenging to accurately estimate the allelic base rate for sex chromosomes (that is, the overall proportion of female embryos), we excluded these from our analysis. Finally, windows in each cross were filtered by requiring that the mean allelic total count (that is, the sum of reads that could be assigned to either allele) across cells was no smaller than 0.1 (Additional file 1: Fig. S8d). This resulted in between 8040 and 12,861 peaks per cross and a combined set of 39,530 peaks to be tested for allelic imbalance (Additional file 1: Fig. S8e).

### scDALI analysis of *Drosophila melanogaster* sci-ATAC data

We applied scDALI to all of the 39,530 peaks to test for heterogeneous (scDALI-Het), homogeneous (scDALI-Hom) and either kind of allelic imbalance (scDALI-Joint). Both scDALI-Het and scDALI-Joint used the 8-dimensional VAE latent space embedding as a cell state representation and a linear kernel function. *P*-values from each test were Benjamini-Hochberg adjusted to control the false discovery rate [52] (FDR). For each of the 415 sites with evidence for cell state-specific allelic imbalance (scDALI-Het *P* < 0.1 FDR), we estimated allelic rates using scDALI. Depending on the number of covered cells for each peak, all models were trained with a maximum of 1000 inducing points.

To compute transcription factor activity scores, deviations in accessibility were calculated with chromVAR v1.10.0 [32] for a set of 65 curated *Drosophila* motifs from [4]. The *Z*-score corrected deviations were used to calculate the Pearson correlation with the estimated allelic rates.

To identify variable lineages for each of the 415 peaks with heterogeneous imbalance, we used scDALI-Het with lineage-specific cell state kernels. Specifically, for each lineage we used a block diagonal kernel matrix, where entry *i*, *j* was set to 1 if both cells *i* and *j* were associated with that lineage and 0 otherwise. This allowed us to test for differences in the mean allelic rate for a particular lineage compared to the mean of all other cells. The combined *P*-values for all lineages were adjusted for multiple testing using the Benjamini-Hochberg correction [52].

Lastly, we employed scDALI-Het to test for changes in allelic imbalance across development in the muscle lineage using the temporal ordering inferred by the VAE model. To capture non-linear effects, we applied a polynomial basis transform. Specifically, we constructed a matrix *E*_*Time*_ with entries (*E*_*Time*_)_*ij*_ = *t*_*i*_^*j* − 1^, *j* ∈ {1, 2, 3}, where *t*_*i*_ ∈ [0, 1] denotes the estimated time for cell *i* in the muscle lineage. We then applied scDALI assuming a linear cell-state kernel *K* = *E*_*Time*_*E*_*Time*_^*T*^. *P*-values were adjusted using the Benjamini-Hochberg correction [52].

### Evaluation of scDALI on simulated data

We simulated allele-specific counts from the scDALI model (Eq. (1–3)) using observed allelic total counts and inferred cell state representations (VAE embedding and Leiden clusters derived from the VAE embedding) from real sci-ATAC-seq data of developing *Drosophila melanogaster* embryos (cross F1-DGRP-712). All simulations kernels were linear, that is *K*_*VAE*_ = *E*_*VAE*_*E*_*VAE*_^*T*^ and *K*_*Cluster*_ = *E*_*Cluster*_*E*_*Cluster*_^*T*^ where *E*_*VAE*_ and *E*_*Cluster*_ denote the 8-dimensional VAE embedding and one-hot encoding of 24 Leiden clusters, respectively.

We assessed the degree of extra-binomial variation present in the data, by fitting a basic Beta-Binomial model to the observed allele-specific counts (10,220 cells and 12,861 peaks) using no additional cell state information (Additional file 1: Fig. S1a). Based on the histogram of estimated values, we ran all simulations at two different levels of overdispersion *θ* ∈ {2, 5}.

We first assessed the calibration of scDALI when testing for heterogeneous effects (scDALI-Het). As scDALI is intended to leverage multi-dimensional cell-state representations, we analyzed the effect of testing an increasing number of cell-state dimensions for different numbers of cells. We considered two baseline candidates: a one-way ANOVA test, comparing allelic rates between cell clusters as well as a linear model incorporating cell-state covariates as fixed effects (likelihood-ratio test, OLS-LRT). Both alternatives were fitted to empirical allelic rates. All three tests used the observed Leiden clustering as a cell-state representation. We simulated data from a model assuming no heterogeneous imbalance, varying the number of clusters (cell-state dimensions) while keeping the number of simulated cells constant. We considered four different sample sizes: 250, 500, 1000, and 5000 cells per peak with non-zero allelic measurements. All experiments were performed for 1000 peaks. We computed the average inflation factor *log*_10_(*median P*)/*log*_10_(0.5) (Additional file 1: Fig. S1d) across 25 different random initializations, finding the OLS-LRT to produce inflated *p*-values for a large number of cell-state dimensions relative to the sample size. This is consistent with results on multiple-degrees-of-freedom tests reported previously [21]. We therefore excluded the OLS-LRT from further simulation experiments.

We verified the uniform distribution of *p*-values for all three scDALI models and the one-way ANOVA when simulating data from their respective null models (1000 peaks and 5000 cells randomly sampled from the full data), considering different levels of pervasive (cell state independent) effects (*α* ∼ *N*(0, *ν*^2^), where *ν*^2^ ∈ {0, 0.01, 0.05, 0.1}; Additional file 1: Fig. S1b, c). Here, all models used the 8-dimensional VAE embedding as a cell-state representation. Additionally, we show that a modified version of scDALI-Het using a Binomial (rather than Beta-Binomial) likelihood model will lead to false positive results at relevant levels of overdispersion.

We compared power to detect homogeneous vs. heterogeneous effects for scDALI-Het, scDALI-Hom and scDALI-Joint (Fig. 1c, Additional file 1: Fig. S3a). Let *ρ* ∈ {0, 0.2, 0.4, 0.6, 0.8, 1} denote the relative extent of heterogeneous imbalance and *ν*^2^ ∈ { 0.01, 0.05, 0.1} the total variance explained by allele-specific effects (both heterogeneous and homogeneous). We simulated data from the scDALI model (Eq. (1–3)), where *α* ∼ *N*(0, *ρ* · *ν*^2^) and *σ*^2^ = (1 − *ρ*) · *ν*^2^, using observed total counts and cell-state representations for 5000 cells and 1000 ATAC peaks randomly chosen from the observed data. All three models as well as the simulation procedure were run using the 8-dimensional VAE embedding as a cell-state representation. Statistical power was calculated as the fraction of simulated regions discovered at an *α*-level of 0.05 and averaged across 25 random seeds.

Lastly, we assessed power to detect discrete vs. continuous heterogeneous effects, using a weighted combination of the VAE and Leiden cluster kernels

$$ K=\eta {K}_{Cluster}+\left(1-\eta \right){K}_{VAE} $$

In this scenario we assumed no additional homogeneous effects (*α* = 0) and considered a range of weights *η* and kernel scaling parameters. (*η* ∈ {0, 0.2, 0.4, 0.6, 0.8, 1} and *σ*^2^ ∈ {0.1, 0.05, 0.1}, Fig. 1d, Additional file 1: Fig. S3b). Compared were scDALI-Het using the VAE representation vs. a one-way ANOVA model based on the discrete Leiden clusters. As above, we simulated data for 5000 cells and 1000 peaks and averaged power estimates across 25 random initializations.

### Analysis of allelic imbalance in population-scale iPSC data

Total gene expression counts for all genes and allele-specific quantifications for 4470 previously identified SNP-gene pairs (4422 eQTL lead variants) were obtained as described in the primary publication [5]. Briefly, reads were initially mapped to reference and alternative alleles for each heterozygous SNP in every cell and subsequently assigned relative to the genotype of each chromosome using known phasing information. Allele-specific read counts were aggregated at the gene level, by summing up the counts for each SNPs contained in exonic regions. Finally, for each eQTL (gene-SNP pair), gene-level allele-specific counts were interpreted relative to the eQTL variant to obtain a consistent definition of ASE across cells from different donors that were heterozygous for that variant. SNP-gene pairs were filtered by requiring at least 50 cells with nonzero allele-specific counts, leading to 3966 pairs to be tested using scDALI. We performed principal component analysis (PCA) of total gene expression counts from 34,254 cells and used the leading *k* principal components (PCs) and a linear kernel function to define cell state kernels. We chose *k* = 1 to focus on time-specific allelic imbalance (see also Additional file 1: Fig. S1) while *k* = 10 was used to model more general cell-state effects.

To assess the effect of donor-specific effects on heterogeneous allele-specific expression, we permuted the leading 10 PC coordinates among cells from the same donor. We then compared two implementations of scDALI-Het that either did or did not account for the donor background using a one-hot-encoded representation of the donor identities for each cell as an additional covariate matrix. Additionally, we compared the difference in the number of discoveries for each model when using cell-state kernels based on 1, 2, 3, 4, 5, 10, 15, and 20 (unpermuted) PCs.

## Supplementary Information

**Additional file 1: Supplementary Information and Figures.**

**Additional file 2: Supplementary Methods.**

**Additional file 3: Supplementary Table S1.**

**Additional file 4.** Review history.

## Data Availability

All raw sequencing data from the *Drosophila* F1 study have been submitted to the EMBL-EBI ArrayExpress database (https://www.ebi.ac.uk/arrayexpress/) and are available under accession number E-MTAB-10240 [53]. Processed data, including the sciATAC peaks per genotype and stage, can all be downloaded from http://furlonglab.embl.de/data. The processed iPSC scRNA-seq data is available on zenodo (https://zenodo.org/record/3625024#.YcM1GS8w0eb) and all HipSci genotyping data can be accessed under http://www.hipsci.org. A Python implementation of the scDALI framework including the VAE model for cell state inference is available under the BSD-3 license from https://github.com/PMBio/scdali [54]. Code to reproduce the specific analyses is available from https://github.com/PMBio/scdali_analyses and was deposited on zenodo (https://zenodo.org/record/5710797) [55].
