# Supplementary material for: scDALI: modeling allelic heterogeneity in single cells reveals context-specific genetic regulation
Source: Genome Biol. 2022 Jan 6;23:8. doi: 10.1186/s13059-021-02593-8 (PMC8734213; doi:10.1186/s13059-021-02593-8)
Supplement: Supplementary file 1 — Additional file 1: Supplementary Information and Figures. [file 13059_2021_2593_MOESM1_ESM.pdf]

scDALI: modelling allelic heterogeneity in single cells reveals context-specific genetic regulation

Additional file 1: Supplementary Information and Figures

Supplementary tables **2**

Supplementary figures **3**

Supplementary tables

Table S1 | Summary of scDALI test results for *Drosophila* F1 data provided as a separate data file (additional_file_3.xlsx). Contained are peaks that were significant at 0.1 FDR for any scDALI test. The fields are as follows:

chr	Peak coordinates.
start	
end	
n_cells	Number of cells with nonzero counts.
rate	Empirical rate across all cells (sum of reads mapping to the maternal haplotype divided by the total number of reads).
qdiff10	Effect size for heterogeneous allelic imbalance (**Methods**). Only provided for peaks with scdalihet_p_adj < 0.1.
scdalihet_p	scDALI-Het p-value.
scdalihom_p	scDALI-Hom p-value.
scdalijoint_p	scDALI-Joint p-value.
scdalihet_p_adj	Benjamini-Hochberg adjusted scDALI-Het p-value.
scdalihom_p_adj	Benjamini-Hochberg adjusted scDALI-Hom p-value.
scdalijoint_p_adj	Benjamini-Hochberg adjusted scDALI-Joint p-value.
scdalijoint_rho	scDALI-Joint ρ(estimated relative extent of heterogeneous imbalance, **Methods**).

Supplementary figures

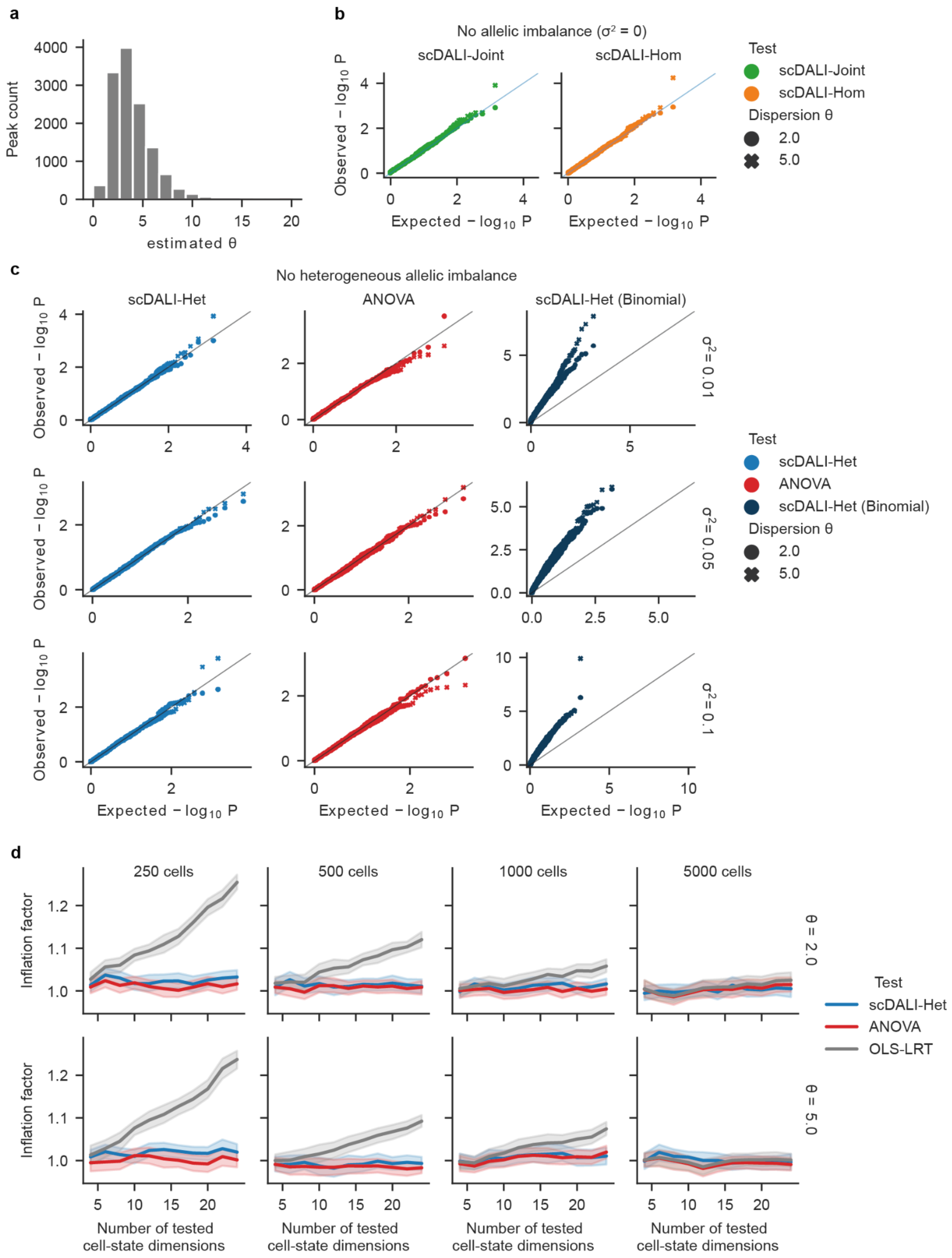

a
Peak count
estimated θ
b
No allelic imbalance (σ² = 0)
scDALI-Joint
scDALI-Hom
Observed − log10 P
Expected − log10 P
Test
scDALI-Joint
scDALI-Hom
Dispersion θ
2.0
5.0
c
No heterogeneous allelic imbalance
scDALI-Het
ANOVA
scDALI-Het (Binomial)
σ² = 0.01
σ² = 0.05
σ² = 0.1
Test
scDALI-Het
ANOVA
scDALI-Het (Binomial)
Dispersion θ
2.0
5.0
d
250 cells
500 cells
1000 cells
5000 cells
Inflation factor
θ = 2.0
θ = 5.0
Number of tested cell-state dimensions
Test
scDALI-Het
ANOVA
OLS-LRT

Figure S1 | Assessment of statistical calibration of scDALI and alternative methods on simulated data. (a) Histogram of estimated overdispersion parameters for individual peaks of chromatin accessibility for F1 cross F1-DGRP-712 (10,220 cells and 12,861 peak), using a Beta-Binomial model without any cell state-specific information. **(b, c)** QQ plots to assess empirical calibration of alternative tests on data simulated from a Beta-Binomial model, with constant mean sampled from a Normal distribution with mean zero and variance σ^2(rows) and two alternative levels of overdispersion θ. Non-zero values of σ^2 correspond to homogeneous but no heterogeneous imbalance. Shown are results from 1,000 tested peaks and 5,000 cells randomly sampled from the chromatin accessibility data for F1 cross F1-DGRP-712, with total counts corresponding to observed data as in **a**. scDALI-Joint and scDALI-Het models were provided with a continuous cell-state kernel derived from the latent space of a VAE model trained on real data (**Fig. S7, Methods**). Considered were scDALI-Hom, scDALI-Joint, scDALI-Het, scDALI-Het (Binomial), a model that does not account for extra-binomial dispersion and an ANOVA model to test for differences in mean allelic rates between discrete Leiden clusters inferred based on the VAE embedding. **(d)** Statistical calibration as a function of the number of tested environments (Leiden clusters) for varying numbers of cells with nonzero total counts. Data were simulated from a null model assuming no heterogeneous allelic imbalance. Shown are P-value inflation factors $\log_{10}(\text{median}(P))/\log_{10}(0.5)$ for a likelihood ratio test in a linear regression model on empirical allelic rates (OLS-LRT), scDALI-Het and an ANOVA test. When testing a large number of cell-states compared to the sample size, the likelihood ratio test produces inflated test statistics, while both scDALI-Het and the ANOVA test remain calibrated across all scenarios.

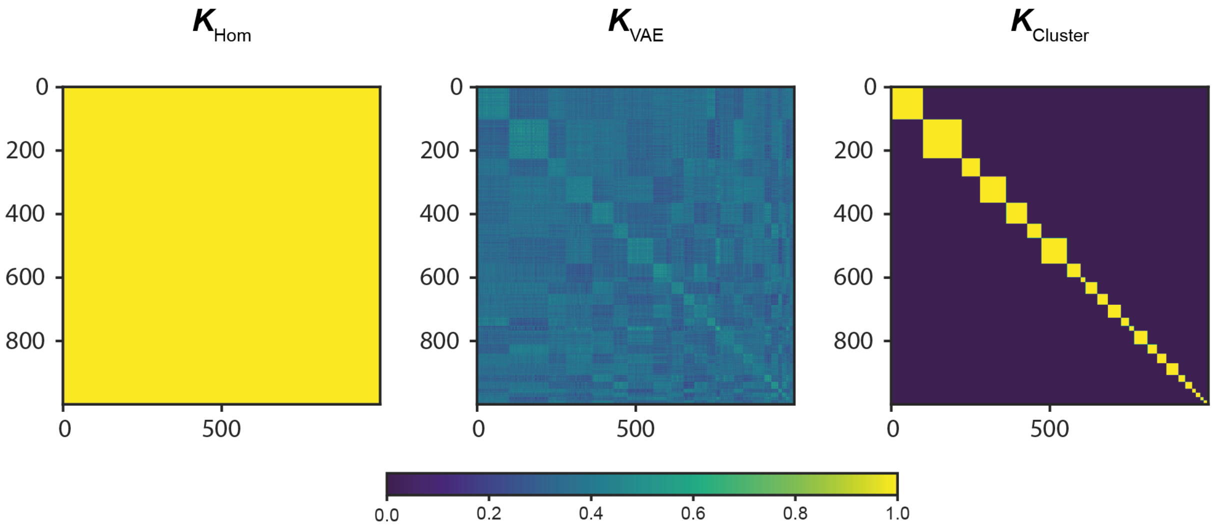

Figure S2 | Cell-state kernels for the simulation procedure. Kernel matrices for simulation experiments. Left: Homogeneous kernel (matrix of ones). Middle: Cell state kernel based on the inner product similarity in the VAE cell state space for 10,220 cells from cross F1-DGRP-712 (**Methods, Fig. S7a**). Right: Discrete cluster kernel; a block diagonal matrix indicating Leiden cluster membership (N=25 clusters, **Fig. S7b**). All kernels are subsampled to 1,000 cells for visualization purposes.

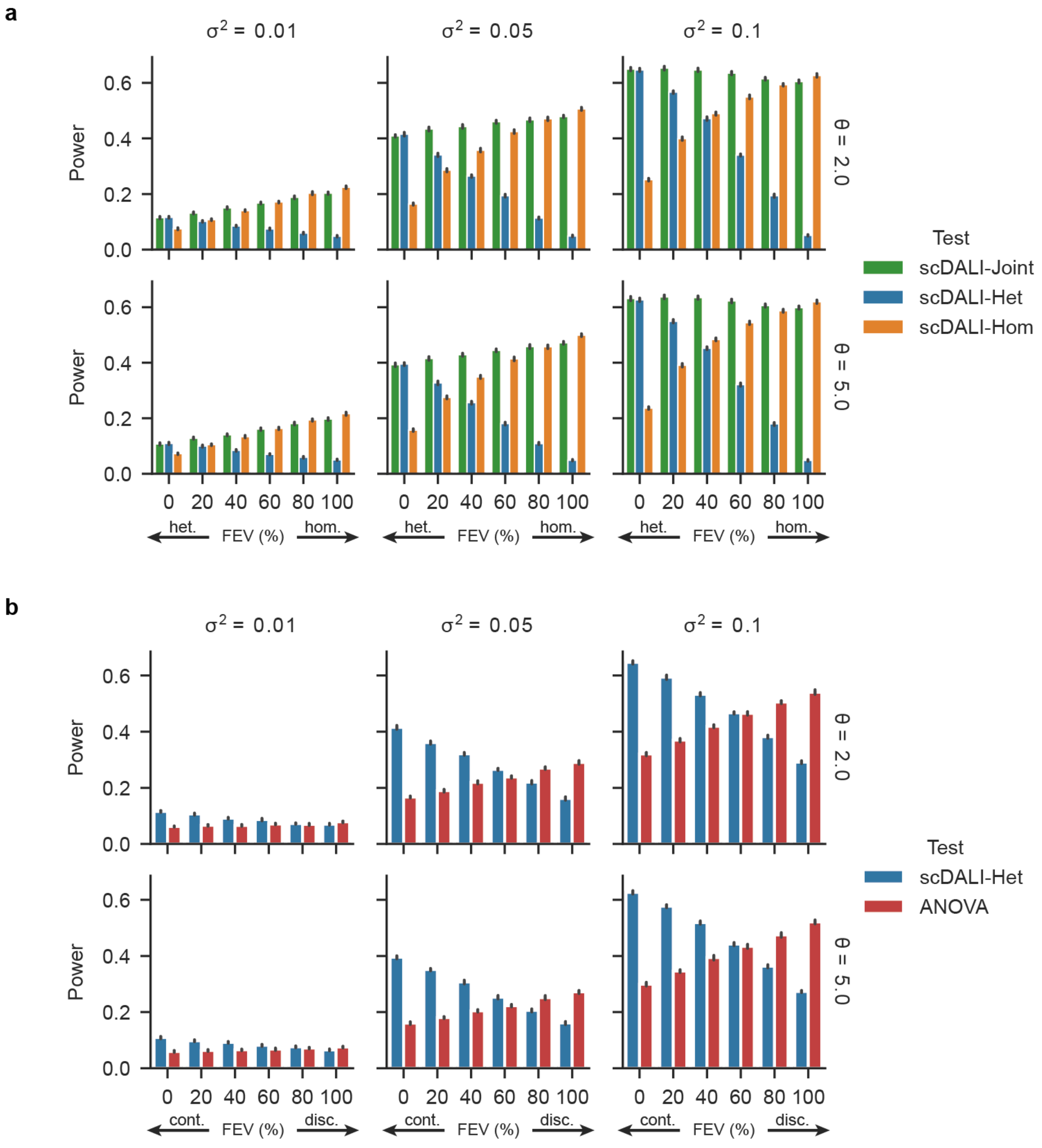

Figure S3 | Additional results from the power assessment on simulated data. Alternative counts were generated from the scDALI model, when considering different levels of variance explained by the simulation kernel (σ^2, columns) for two levels of overdispersion (θ, rows). Results in **Fig. 1b-e** correspond to $\sigma^2 = 0.05$. **(a)** Varying the extent of simulated heterogeneous vs. homogeneous allelic imbalance (**Methods**). **(b)** Interpolating between simulated continuous cell states (VAE embedding based on real data) and discrete cell states (Leiden clustering of VAE cell states, 24 clusters). The x-axis shows the fraction of explained variance of allele-specific effects (FEV, **Methods**). Barplots indicate the fraction of peaks discovered by scDALI-Het, scDALI-Hom and scDALI-Joint **(a)**, and scDALI-Het vs. a one-way ANOVA testing for differences between the 24 Leiden clusters **(b).** All scDALI-Het and scDALI-Joint models were using the continuous VAE embedding as a cell-state representation.

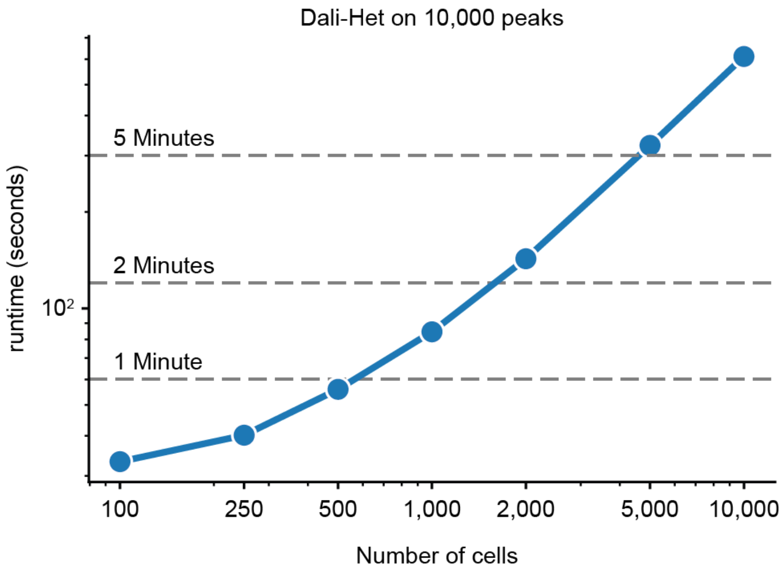

Figure S4 | DALI-Het runtime analysis. Empirical runtimes for 10,000 tests of randomly sampled peaks for cross F1-DGRP-712 for increasing number of cells. DALI-Het scales linearly with the number of cells. Cells with non-zero total allelic counts were sampled with replacement to ensure the same number for each peak. Runtimes were evaluated on a 2018 MacBook Pro with 2,3 GHz Quad-Core Intel Core i5 processor.

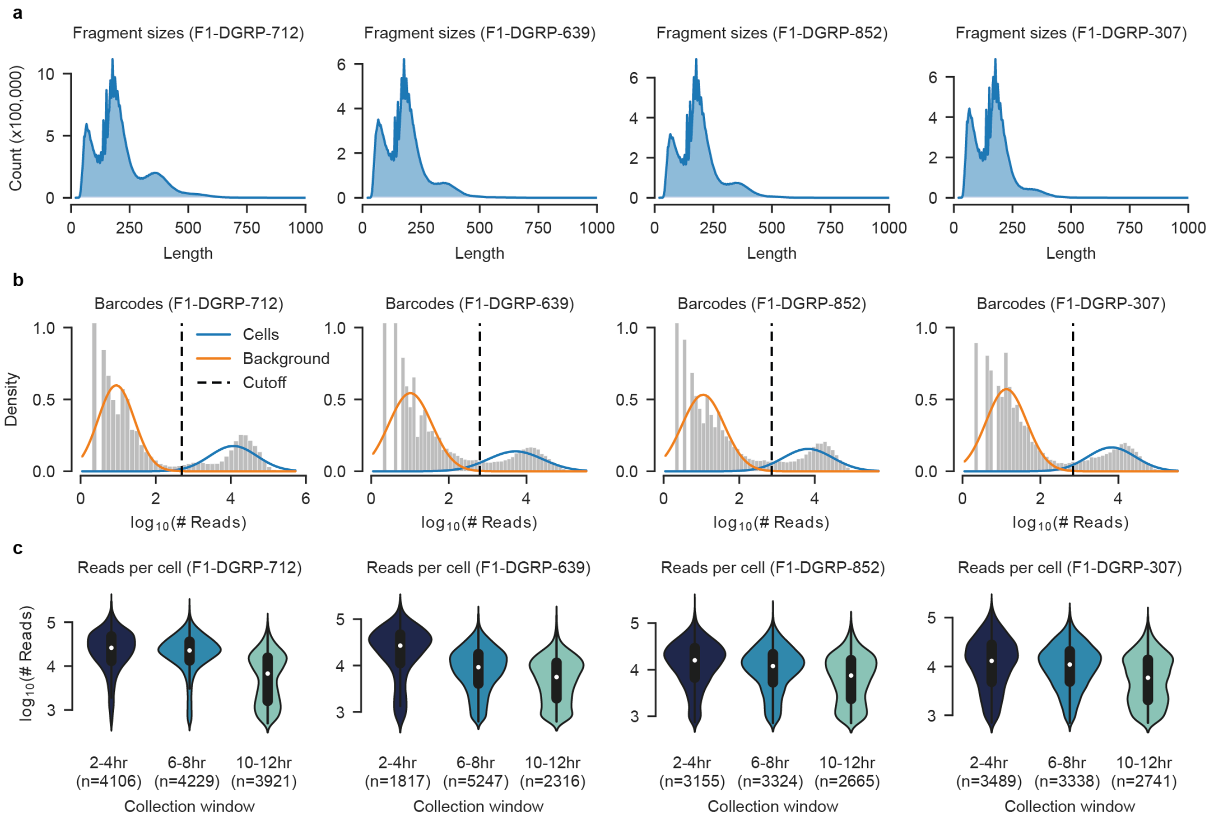

Figure S5 | QC for *Drosophila melanogaster* sci-ATAC-seq data. (a) Fragment size distribution for all four crosses. The multi-modal distribution corresponds to the expected nucleosome banding pattern. **(b)** Distribution of log-total read counts per barcode. A two-component Gaussian mixture model is fitted to discern background signal from genuine cells (orange and blue lines). The dotted line represents the cutoff corresponding to the 95% posterior probability of cells belonging to the foreground mixture component. **(c)** Violin plots showing the read count distribution for cells identified in **b** stratified by the embryo collection window (2-4, 6-8 and 10-12 hours after egg laying).

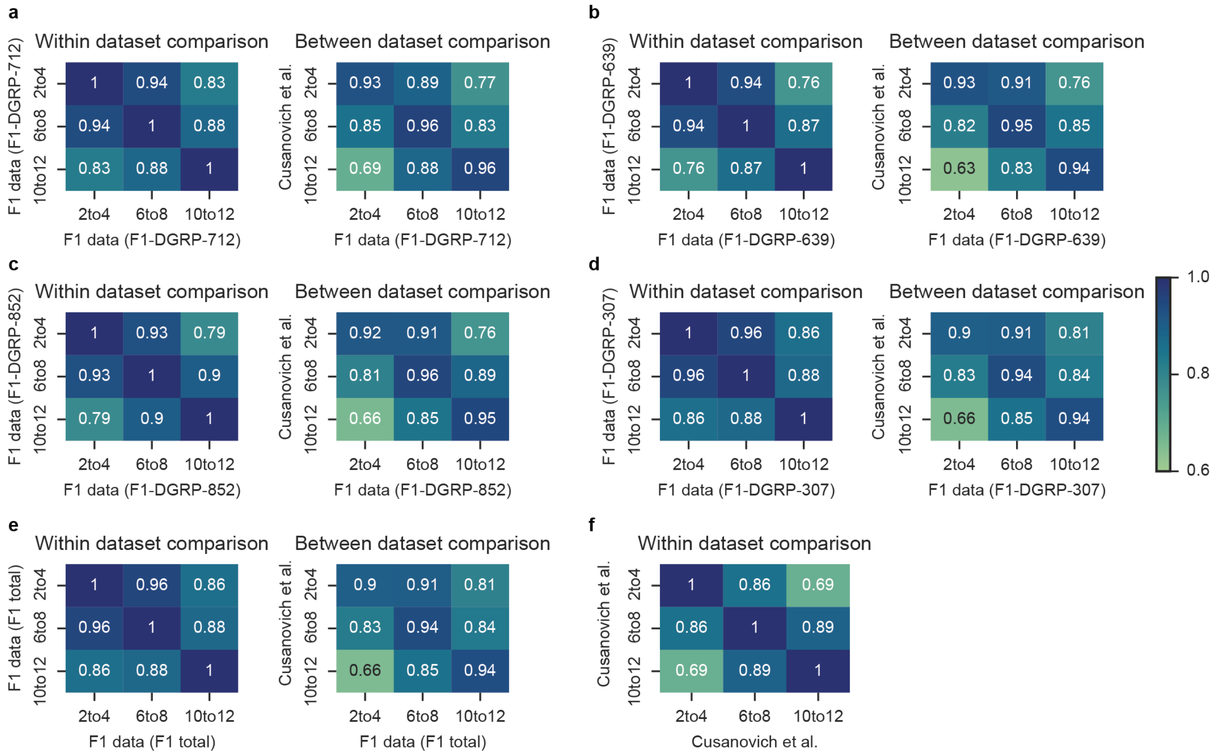

Figure S6 | Comparison with published sci-ATAC data. (a-d) Pearson correlation of pseudo-bulk accessibility profiles across ATAC peaks, both within our dataset as well as compared to published, time-matched sci-ATAC data (Cusanovich et al. 2018) for each cross. Matching timepoints are highly correlated between both datasets. **(e)** Pearson correlation for the combined dataset comprising all four crosses. **(f)** Within dataset comparison for the Cusanovich et al. data.

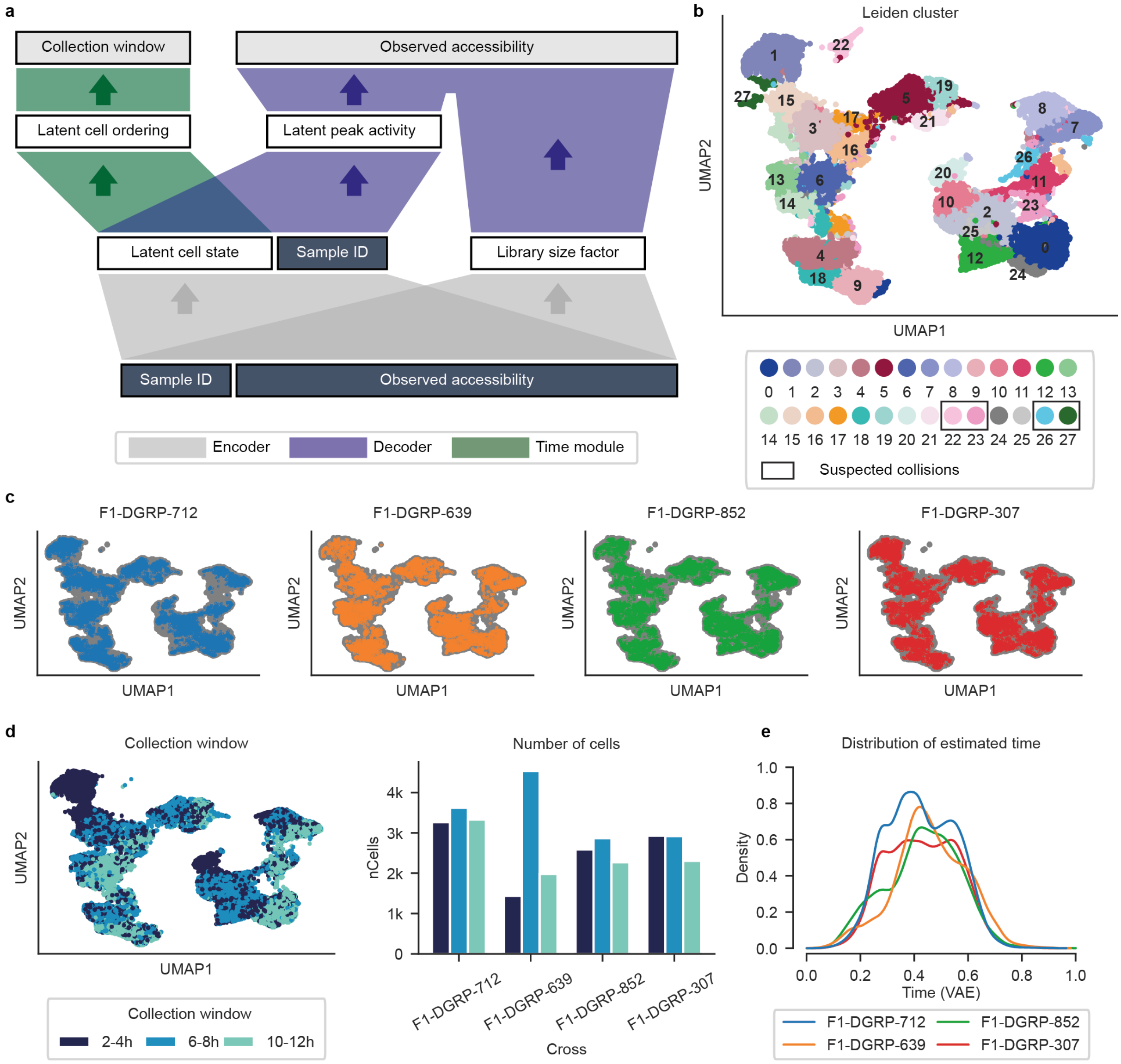

Figure S7 | sci-ATAC Variational autoencoder (VAE). (a) Model architecture. Colors highlight the three major building blocks. An encoder network maps observed (total) accessibility counts into a lower dimensional cell state space and disentangles library size variation and batch/sample identity. A decoder network first maps cell states and sample ids to a latent peak activity score, indicating the relative "openness" of each peak. Latent peak activity scores are then combined with cell-specific size factors to form a size-factor adjusted Bernoulli likelihood for the observed data. A temporal module infers a continuous pseudo-temporal ordering by mapping cell states to discrete ordered timepoints using an ordinal likelihood model. **(b)** 2-dimensional UMAP visualization of the 8-dimensional VAE cell state representation colored by Leiden clusters. Clusters 22, 23, 26 and 27 were suspected to correspond to barcode collisions and later discarded (**Methods**). **(c)** UMAP visualization as in **b** with individual panels highlighting the cells from each cross. The VAE uniformly integrates all four F1 crosses in a joint cell state space. **(d)** Left: UMAP embedding colored by the (observed) time point of embryo collection, revealing a coarse-grained temporal trajectory. Right: Number of cells associated with each embryo collection window after filtering cells with very high (more than 99% quantile) or very low counts (less than 10% quantile) in each timepoint. **(e)** Distribution of estimated continuous time for individual cells as inferred by the VAE model for each cross.

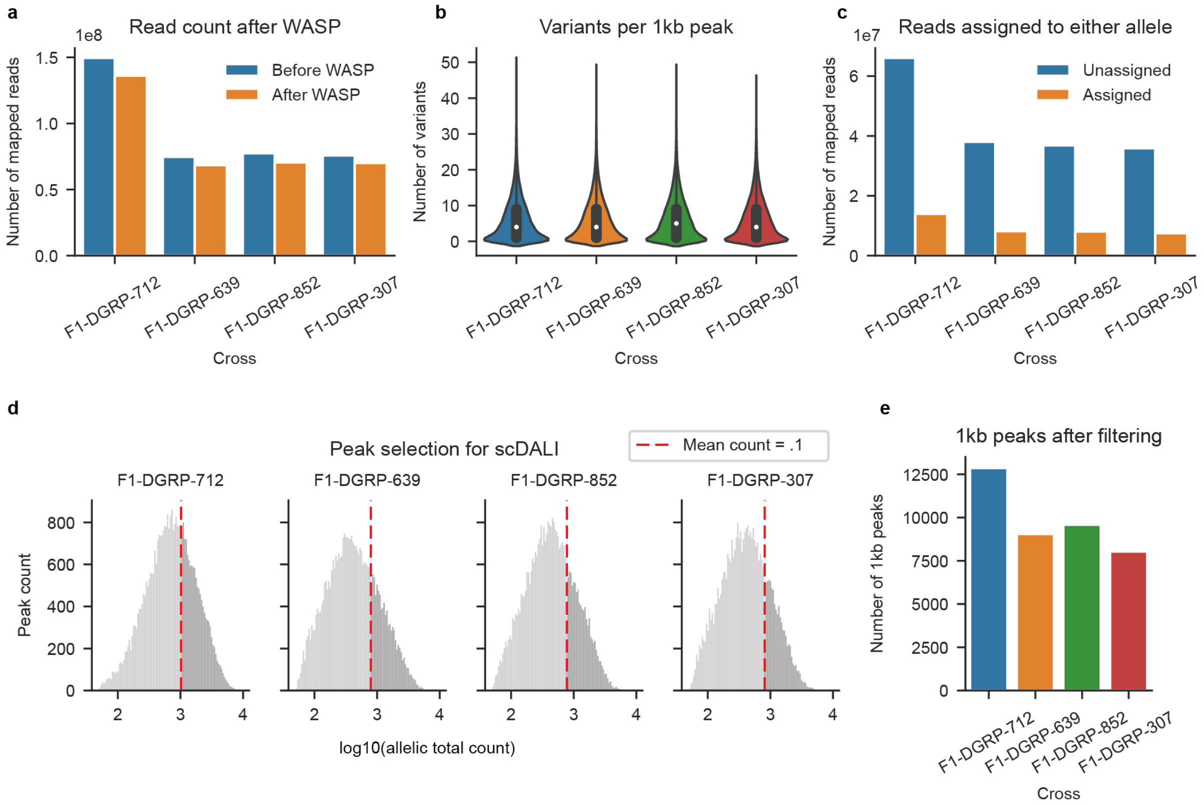

Figure S8 | QC for allele-specific quantifications. (a) Read counts before and after applying the WASP pipeline to reduce reference mapping biases. **(b)** Number of variants across 1kb windows centered on peaks in each cross. **(c)** Number of assigned and unassigned reads for each cross. About 20% of all reads overlapping 1kb windows centered on peaks can be assigned to either allele. **(d)** Peak selection for the scDALI tests. Shown are histograms of the allelic total read counts (total number of reads that can be assigned to either allele). The dotted red line indicates a mean allelic total count per 1kb peak of .1, which was used as a filtering cutoff. **(e)** Number of 1kb peaks per cross after filtering. The combined set of 39,530 peaks was tested for allelic imbalance using scDALI.

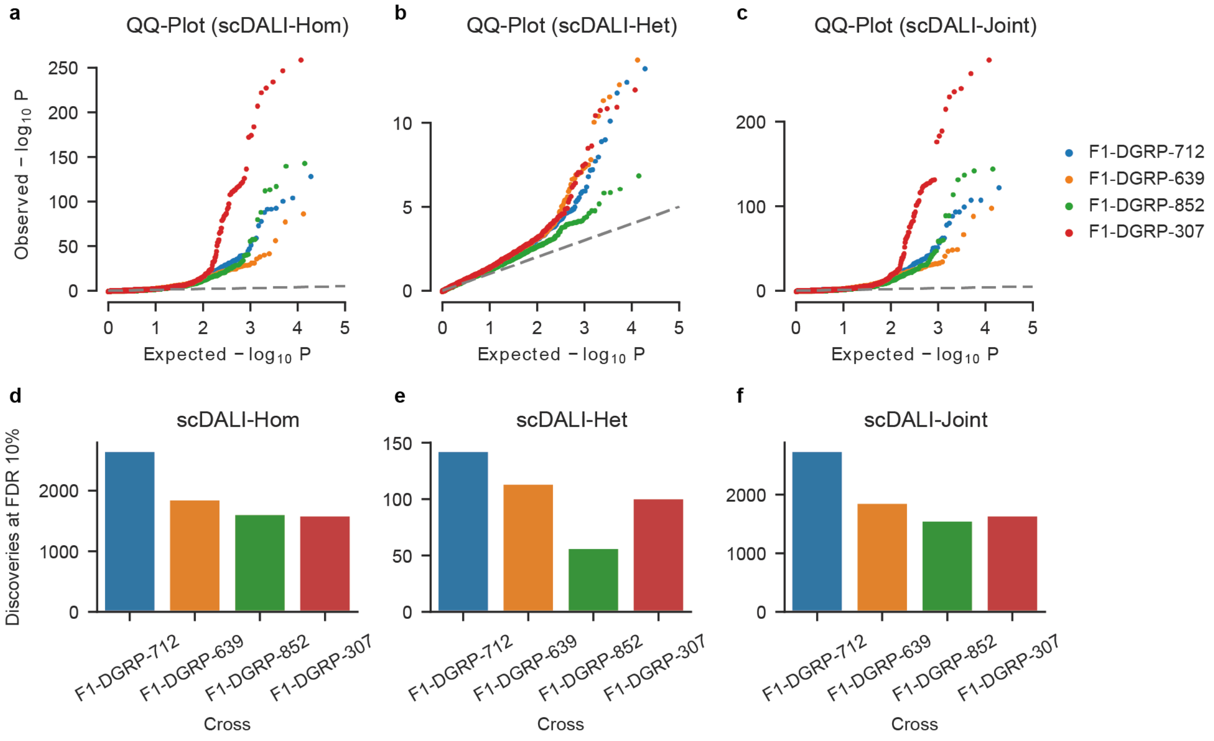

Figure S9 | DALI discoveries. (a-c) QQ Plots for scDALI-Hom, scDALI-Het and scDALI-Joint applied to allele-specific ATAC-seq of developing Drosophila embryos. Colors indicate four different crosses. **(d-f**) Number of discoveries for each test stratified by cross.

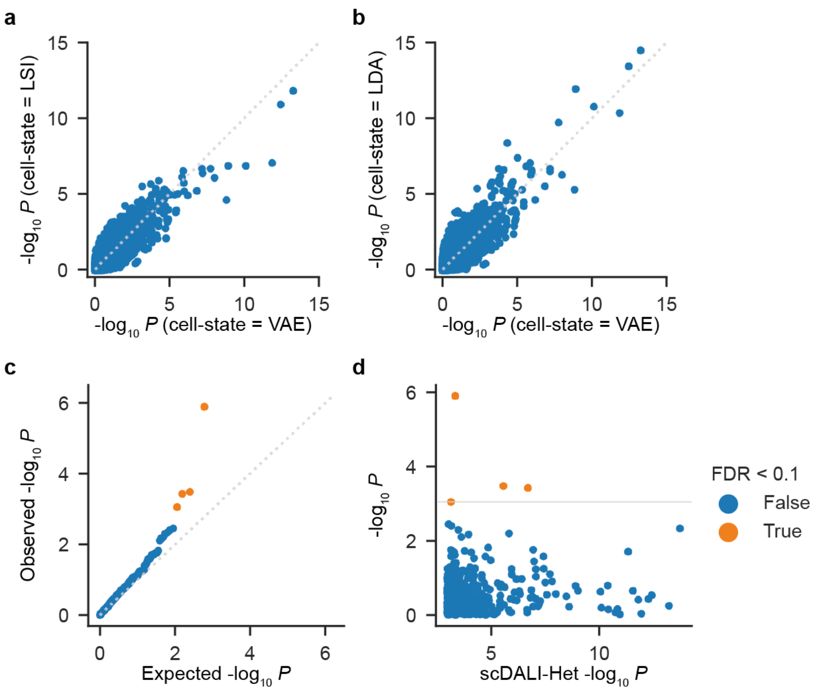

Figure S10 | scDALI diagnostics on real data. (a, b) Comparison of negative log10 P-values when using alternative cell-state representations for scDALI-Het (data from F1 cross F1-DGRP-712, 10,220 cells and 12,861 peaks). Results for the 8-dimensional VAE embedding used throughout the main analysis are compared with **(a)** latent semantic indexing (LSI; leading components 2-20, the first component was excluded due to high correlation with the total number of read counts per cell, **Methods**) and **(b)** latent dirichlet allocation (implemented in cisTopic, 50 topics). **(c, d)** Assessment of associations between allelic imbalance and total accessibility for individual peaks with evidence for heterogeneous allelic imbalance (**Methods**). Shown are results for 415 peaks identified using scDALI-Het. **(c)** QQ-plot of observed and expected p-values, for an association test between total counts and allelic imbalance for each peak. **(d)** Scatter plot of negative log10 p-values between the association test as in **c** and scDALI-Het. Out of 415 peaks with significant cell-state-specific allelic imbalance only 4 show evidence for an association between total and allele-specific counts (FDR < 0.1).

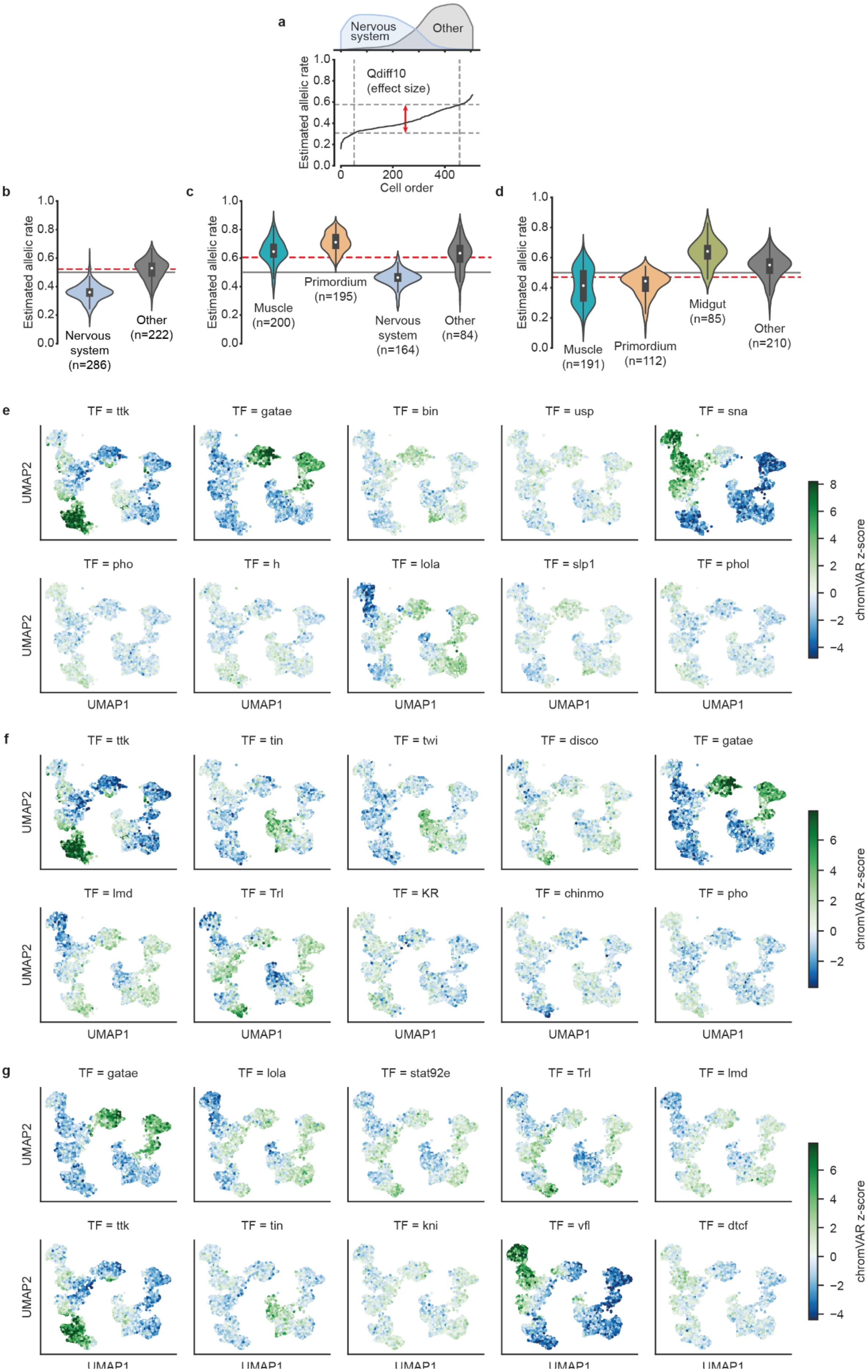
a
Nervous system
Other
Qdiff10
(effect size)
Estimated allelic rate
Cell order
b
Nervous system (n=286)
Other (n=222)
c
Muscle (n=200)
Primordium (n=195)
Nervous system (n=164)
Other (n=84)
d
Muscle (n=191)
Primordium (n=112)
Midgut (n=85)
Other (n=210)
e
TF = ttk
TF = gatae
TF = bin
TF = usp
TF = sna
TF = pho
TF = h
TF = lola
TF = slp1
TF = phol
f
TF = ttk
TF = tin
TF = twi
TF = disco
TF = gatae
TF = lmd
TF = Trl
TF = KR
TF = chinmo
TF = pho
g
TF = gatae
TF = lola
TF = stat92e
TF = Trl
TF = lmd
TF = ttk
TF = tin
TF = kni
TF = vfl
TF = dtcf
UMAP1
UMAP2
chromVAR z-score

Figure S11 | Downstream analysis of cell-state-specific effects for three example peaks. (a) The difference between the top and bottom 10% quantiles of estimated allelic rates by cell defines a measure of effect size for heterogeneous allelic imbalance. **(b-d)** Violin plots showing the distribution of estimated allelic rates in selected lineages for each of the three example regions depicted in **Fig. 3 a-j**. Solid grey lines indicate allelic balance (rate = 0.5), while the dotted red line shows the estimated mean allelic rate across all cells. **(e-g)** chromVAR transcription factor activity scores (z-scores) based on the total accessibility of associated motives for TFs shown in **Fig. 3 c, g, j**.

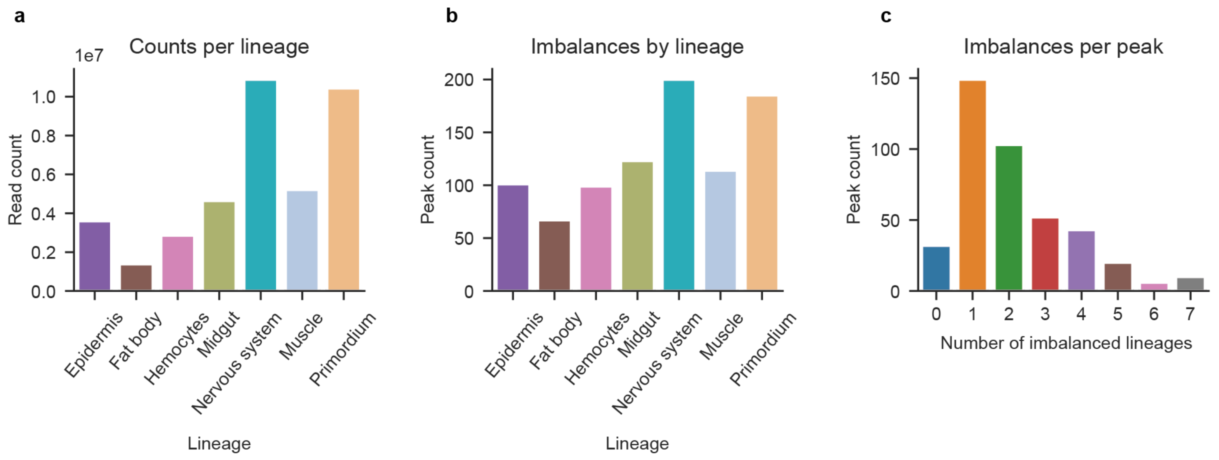

Figure S12 | Additional results from the by lineage analysis. Considered were peaks with significant heterogeneous imbalances (adjusted scDALI-Het P < 0.1) **(a)** Total read count per lineage. **(b-c)** scDALI-Hom applied to discover (homogeneous) allelic imbalances per lineage. **(b)** Number of peaks showing allelic imbalances (FDR < 0.1). The number of discoveries strongly resembles the total read count per lineage in **a.** **(c)** Number of imbalances per peak. The majority of peaks with heterogeneous allele-specific effects show imbalances in one or two lineages.

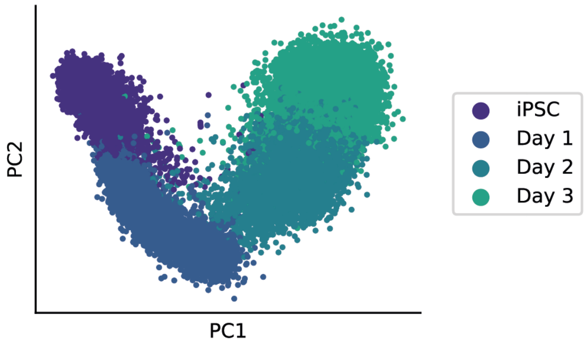

Figure S13 | scRNA-seq of differentiating iPS cells. Principal component analysis (PCA) of total gene expression counts for 34,254 cells. Shown are cell embeddings using the first two principal components, colored by the collection timepoint.
