# Supplementary material for: scDALI: modeling allelic heterogeneity in single cells reveals context-specific genetic regulation
Source: Genome Biol. 2022 Jan 6;23:8. doi: 10.1186/s13059-021-02593-8 (PMC8734213; doi:10.1186/s13059-021-02593-8)
Supplement: Supplementary file 2 — Additional file 2: Supplementary Methods. [file 13059_2021_2593_MOESM2_ESM.pdf]

scDALI: modelling allelic heterogeneity in single cells reveals context-specific genetic regulation

Additional file 2: Supplementary Methods

T. Heinen, S. Secchia, J. P. Reddington, B. Zhao,
E. E. M. Furlong, O. Stegle

Contents

1 The scDALI model **3**

1.1 A Beta-Binomial model for allelic imbalance . . . 3

1.1.1 Parameter estimation from single-cell data . . . 3

1.2 Capturing heterogeneous imbalance . . . 4

1.3 Statistical significance testing . . . 4

1.3.1 scDALI-Het . . . 5

1.3.2 scDALI-Joint . . . 6

1.3.3 scDALI-Hom . . . 7

2 Cell state variational autoencoder model **8**

2.1 Variational inference and encoder networks . . . 8

2.2 Practical considerations . . . 8

1 The scDALI model

scDALI extends the frequently used Beta-Binomial observation model for allele-specific counts in bulk-sequencing data [1, 2, 3, 4], by accounting for cell state-specific effects.

We model the relationship between cell states and allelic rates for a given genomic region using a hierarchical Bayesian model. For cells $i = 1, \ldots, n$, let a_i be the number of reads mapping to the the maternal haplotype and d_i be the total number of reads (for ease of notation, we omit the dependence on the region of interest). We will initially assume that the measurements for each cell correspond to independent trials, and introduce the basic Beta-Binomial model. In section 1.2 we then discuss how the scDALI framework can be used to capture cell state specific covariances. While our terminology will focus on chromatin accessibility data, the same modeling principles generalize to other count-based single-cell assays including single-cell RNA-seq.

1.1 A Beta-Binomial model for allelic imbalance

Assume we are studying the allele-specific accessibility of a particular region among cells from a homogeneous population. That is, all cells share the same underlying allelic rate p, the average accessibility of the maternal haplotype. Then, given the observed total number of reads d_i, we can regard a_i as a draw from a binomial distribution

$$a_i \,|\, d_i \sim \mathrm{Bin}(d_i, p). \tag{1}$$

Real data, however, shows more variability than is to be expected under a binomial model. This is because cellular populations are rarely entirely homogeneous (e.g. due to cell cycle effects) and allele-specific counts are affected by additional technical and biological sources of variation. One can account for this by making p itself a random variable. A common choice is the Beta distribution [1, 2, 3, 4]

$$p \sim \mathrm{Beta}(\theta^{-1}\mu, \theta^{-1}(1-\mu)), \tag{2}$$

leading to a compound distribution with closed-form density. Here, μ denotes the mean allelic rate and θ is an overdispersion parameter modulating the amount of extra-binomial variance. For small θ, the Beta-Binomial distribution approaches a Binomial distribution. Conversely, when θ is large, draws will resemble a Bernoulli distribution with counts coming almost exclusively from either allele.

1.1.1 Parameter estimation from single-cell data

While the Beta-Binomial distribution does not allow for closed-form maximum-likelihood estimates, efficient numerical optimization algorithms are available [5]. Estimating parameters from bulk sequencing data is challenging, as the number of replicates is often limited. To lower the estimation uncertainty,

further assumptions are usually required, such as a shared mean-variance relationship between genomic regions [1]. Single-cell sequencing assays, on the other hand, provide large numbers of cells / samples and in principle allow for direct estimation of θ and μ separately for each region. Note that we are still assuming that the allelic rates in different cells are independent and identically distributed (i.i.d.). This assertion, however, is unlikely to hold in practice as molecular traits are correlated across cell types and states. In the next section, we therefore extend eq. (1-2) to account for cellular heterogeneity.

1.2 Capturing heterogeneous imbalance

Building on Gaussian process (GP) regression [6], we capture cell state-specific allelic variation by introducting a latent n-dimensional Gaussian variable

$$\mathbf{u} \sim \mathcal{N}(\mathbf{1} \cdot \alpha + \mathbf{X}\boldsymbol{\beta}, \sigma_{het}^2 \mathbf{K}). \tag{3}$$

Here, $\mathbf{u}$ denotes the vector of allelic rates on the logit scale, which is coupled to the Beta-Binomial observation model for allelic counts introduced in section 1.1 as follows

$$\mu_i = g^{-1}(u_i) \tag{4}$$

$$a_i \,|\, \mu_i, d_i \sim \text{Beta-Binomial}(\theta^{-1}\mu_i, \theta^{-1}(1 - \mu_i)), \tag{5}$$

where g is the logit link function, $g(x) = \log(\frac{x}{1-x})$. The constant offset α models homogeneous imbalance (here $\alpha = 0$ corresponds to an allelic rate of 1/2), while cell state covariances are encoded in a kernel matrix $\mathbf{K} \in \mathbb{R}^{n \times n}$. The scaling parameter σ_{het}^2 determines the total variance explained by cell state-specific effects, while the fixed effect $\boldsymbol{\beta}$ captures the influence of additional (optional) covariates $\mathbf{X}$ such as batch or sample identity.

1.3 Statistical significance testing

The scDALI model specified in eq. (3-5) uses two parameters to capture allelic imbalances: The fixed effect α encodes *homogeneous* imbalance affecting all cells equally across the state space, while the variance component σ_{het}^2 determines the magnitude of cell state-specific, i.e. *heterogeneous* effects. We consider three different null and alternative hypothesis, for which efficient score-based tests are derived in the following sections:

scDALI-Hom Presence of *homogeneous* allelic imbalance (section 1.3.3)

$$H_0^{hom} : \alpha = 0 \text{ vs. } H_1^{hom} : \alpha \neq 0. \tag{6}$$

scDALI-Het Presence of *heterogeneous* allelic imbalance (section 1.3.1)

$$H_0^{het} : \sigma_{het}^2 = 0 \text{ vs. } H_1^{het} : \sigma_{het}^2 > 0. \tag{7}$$

scDALI-Joint Presence of either *homogeneous* or *heterogeneous* allelic imbalance (section 1.3.2)

$$H_0^{joint} : \alpha = 0 \text{ and } \sigma_{het}^2 = 0 \text{ vs. } H_1^{joint} : \alpha \neq 0 \text{ or } \sigma_{het}^2 > 0. \tag{8}$$

1.3.1 scDALI-Het

To test for heterogeneous allelic imbalance, we need to assess whether $\sigma_{het}^2 > 0$. One possibility is to use a likelihood ratio test (LRT) [7]. However, the LRT requires to fit parameters for both the null and alternative models which is computationally expensive. Furthermore, the null hypothesis places σ_{het}^2 on the boundary of the parameter space, and will result in a likelihood-ratio test statistic that does not follow a chi-square distribution asymptotically. Instead, we implement an efficient score-based testing procedure [8, 9, 10, 11], scaling linearly with the number of cells if the cell state kernel $\mathbf{K}$ is of low rank.

The derivation of the score statistic for the model defined in Eq. (4-5) largely follows [9] and [10]. Consider the maternal allelic ratios $r_i = a_i/d_i$ under the model defined in (3-5). Conditioned on the vector of random effects $\mathbf{u}$, the r_i are independently following a Beta-Binomial distribution with

$$\mathbb{E}[r_i \mid u_i] = \mu_i \tag{9}$$

$$\mathrm{Var}(r_i \mid u_i, d_i) = V(\mu_i, d_i) = \frac{1}{d_i}\mu_i(1-\mu_i)\frac{\theta^{-1}+d_i}{\theta^{-1}+1}. \tag{10}$$

Let $(\hat{\alpha}_0, \hat{\boldsymbol{\beta}}, \hat{\theta}_0)$ be a maximum-likelihood estimate of $(\alpha, \boldsymbol{\beta}, \theta)$ under the null model H_0^{het} and let $\hat{\boldsymbol{\mu}}_0 = g^{-1}(\mathbf{1} \cdot \hat{\alpha}_0 + \mathbf{X}\hat{\boldsymbol{\beta}})$ (link applied element-wise). Similar to [9], we define a null covariance matrix for the Beta-Binomial distribution

$$\mathbf{W}_0 = \mathrm{diag}((V((\hat{\mu}_0)_i, d_i)g'((\hat{\mu}_0)_i)^2)^{-1}) = \mathrm{diag}((\frac{1}{d_i(\hat{\mu}_0)_i(1-(\hat{\mu}_0)_i)}\frac{\hat{\theta}_0^{-1}+d_i}{\hat{\theta}_0^{-1}+1})^{-1}) \tag{11}$$

$$= \mathrm{diag}(d_i(\hat{\mu}_0)_i(1-(\hat{\mu}_0)_i)\frac{\hat{\theta}_0+1}{d_i\hat{\theta}_0+1}), \tag{12}$$

and the projection matrix accounting for the fixed effect

$$\mathbf{P}_0 = \mathbf{W}_0 - \mathbf{W}_0\tilde{\mathbf{X}}(\tilde{\mathbf{X}}^T\mathbf{W}_0\tilde{\mathbf{X}})^{-1}\tilde{\mathbf{X}}^T\mathbf{W}_0. \tag{13}$$

where $\tilde{\mathbf{X}} = \begin{bmatrix}\mathbf{X} & \mathbf{1}\end{bmatrix}$. Following [9], we define the score-based statistic

$$Q = \frac{1}{2}\tilde{\mathbf{r}}_0^T\mathbf{P}_0^T\mathbf{K}\mathbf{P}_0\tilde{\mathbf{r}}_0 \tag{14}$$

where $\tilde{\mathbf{r}}_0$ is the working vector with entries

$$\tilde{\mathbf{r}}_0 = \mathbf{1} \cdot \hat{\alpha}_0 + \mathbf{X}\hat{\boldsymbol{\beta}} + g'(\hat{\boldsymbol{\mu}}_0)(\mathbf{r} - \hat{\boldsymbol{\mu}}_0). \tag{15}$$

One can show that the distribution of Q under the null model H_0^{het} can be approximated by a weighted sum of independent χ_1^2 random variables [12]

$$\sum_k \lambda_k \chi_{1,k}^2, \tag{16}$$

where λ_k are the ordered non-zero eigenvalues of $\frac{1}{2}\mathbf{P}_0^{T/2}\mathbf{K}\mathbf{P}_0^{1/2}$. While computing the eigenvalues of a general $n \times n$ matrix has a computational complexity of $O(n^3)$, a linear kernel $\mathbf{K} = \mathbf{E}\mathbf{E}^T$ typically allows for a much more efficient implementation. Note that for all matrices $\mathbf{A}$, it holds that eigenvalues$(\mathbf{A}^T\mathbf{A}) =$ eigenvalues$(\mathbf{A}\mathbf{A}^T)$ [13]. Therefore, we have

$$\begin{aligned}
\text{eigenvalues}(\frac{1}{2}\mathbf{P}_0^{T/2}\mathbf{K}\mathbf{P}_0^{1/2}) &= \text{eigenvalues}(\frac{1}{2}(\mathbf{E}^T\mathbf{P}_0^{1/2})^T(\mathbf{E}^T\mathbf{P}_0^{1/2})) && (17)\\
&= \text{eigenvalues}(\frac{1}{2}(\mathbf{E}^T\mathbf{P}_0^{1/2})(\mathbf{E}^T\mathbf{P}_0^{1/2})^T) && (18)\\
&= \text{eigenvalues}(\frac{1}{2}\mathbf{E}^T\mathbf{P}_0\mathbf{E}), && (19)
\end{aligned}$$

which can be computed in $O(k^3)$, where the cell state dimensionality k is typically much smaller than the number of cells n. To evaluate the distribution function and obtain p-values we use Davies method [14, 15] as implemented in `limix`[1].

1.3.2 scDALI-Joint

To implement a joint test capable of identifying either heterogeneous or homogeneous allelic imbalance, we adapt the approach originally developed for SKAT-O [10] and more recently extended in [11]. For a given null maternal rate μ_0, let $\alpha_0 = g^{-1}(\mu_0)$. We model homogeneous imbalance α as a second random effect $\alpha \sim \mathcal{N}(\alpha_0, \sigma_{hom}^2)$ such that (3) becomes

$$\mathbf{u} \sim \mathcal{N}(\mathbf{1} \cdot \alpha_0 + \mathbf{X}\boldsymbol{\beta}, \sigma_{hom}^2 \mathbf{1}\mathbf{1}^T + \sigma_{het}^2 \mathbf{K}). \tag{20}$$

Equivalently, one can write

$$\mathbf{u} \sim \mathcal{N}(\mathbf{1} \cdot \alpha_0 + \mathbf{X}\boldsymbol{\beta}, \sigma_{tot}^2[(1-\rho)\mathbf{1}\mathbf{1}^T + \rho\mathbf{K}]) \tag{21}$$

where $\sigma_{tot}^2 = \sigma_{het}^2 + \sigma_{hom}^2$ denotes the total variance explained by allele-specific effects, while $\rho = \sigma_{het}^2/\sigma_{tot}^2 \in [0,1]$ corresponds to relative extent of heterogeneous imbalance (**Fig. 2d**). This allows

[1] `https://github.com/limix/chiscore`

us to reformulate scDALI-Joint as a one-parameter variance component test

$$H_0^{joint} : \sigma^2_{tot} = 0 \text{ vs. } H_1^{joint} : \sigma^2_{tot} > 0 \text{ (scDALI-Joint)}. \tag{22}$$

For given ρ, we can test this hypothesis using the score-based framework described above for scDALI-Het, assuming a modified kernel matrix $\mathbf{K}_\rho = [(1-\rho)\mathbf{1}\mathbf{1}^T + \rho\mathbf{K}]$ and fixing $\alpha = \alpha_0$ in (3). As ρ is unknown in practice, we perform a grid search and combine the resulting p-values:

1. Compute p-values p_{ρ_r} for ρ_r from a pre-defined grid of values in $[0, 1]$. As in [10, 11], we replace the exact Davies method with the modified moment matching approximation [15, 16] to improve computational efficiency when evaluating the null distribution (16).

2. Determine the test statistic $T = \min_r p_{\rho_r}$ and compute the final p-value. Details on the exact form and estimation of the associated null distribution can be found in [11].

1.3.3 scDALI-Hom

As a special case, we can use the scDALI framework to test for homogeneous allelic imbalance by fixing $\rho = 0$ in eq. (21) (corresponding to no cell state-specific effects). Alternatively, one can employ a likelihood ratio test

$$LLR = 2 \cdot \log \frac{p(\mathbf{a} \mid \mathbf{d}, \mu = \hat{\mu}_1, \theta = \hat{\theta}_1)}{p(\mathbf{a} \mid \mathbf{d}, \mu = \mu_0, \theta = \hat{\theta}_0)}, \tag{23}$$

where $p(\cdot)$ denotes the likelihood function for the model described in (1-2), μ_0 is the null allelic rate and all other parameters are estimating using maximum-likelihood. P-values can be calculated using the fact that LLR asymptotically follows a chi-square distribution with one degree of freedom [17].

2 Cell state variational autoencoder model

This section covers the variational approximation and implementation details of the variational autoencoder model which was used to define a cell state representation for the scDALI analysis of developing *Drosophila* embryos. A full description of the generative model can be found in the main text. For a general introduction to variational autoencoder models, see [18].

2.1 Variational inference and encoder networks

Due to the complex, non-linear generative model, evaluating the posterior distribution over latent variables $\mathbf{z}_i, l_i$ is intractable. Instead, we attempt to find approximate posterior distributions using variational inference. That is, we define tractable parametric families of *variational* distributions $q(l_i \,|\, \mathbf{x}_i, c_i)$ and $q(\mathbf{z}_i, \,|\, \mathbf{x}_i, c_i)$ and optimize their parameters by maximizing the following lower bound on the marginal likelihood (the evidence lower bound, or ELBO)

$$\begin{aligned}\log p(x) \geq\; & E_{q(\mathbf{z},\,|\,\mathbf{x},c)q(l\,|\,\mathbf{x},c)}[\log p(\mathbf{x} \,|\, \mathbf{z}, l, c) p(y \,|\, \mathbf{z})] \\ & - \mathrm{KL}[q(\mathbf{z} \,|\, \mathbf{x}, c) \,||\, p(\mathbf{z})] \\ & - \mathrm{KL}[p(l \,|\, \mathbf{x}, c) \,||\, p(l)],\end{aligned} \tag{24}$$

where $\mathrm{KL}(\cdot \,||\, \cdot)$ denotes the Kullback-Leibler divergence and we have made the simplifying assumption that $l_i \,|\, \mathbf{x}_i, c_i$ and $\mathbf{z}_i, \,|\, \mathbf{x}_i, c_i$ are independent (mean-field variational inference). In fact, maximizing the ELBO is equivalent to minimizing the KL divergence between the true (unknown) posterior and the variational approximation [19]. A key idea of the variational autoencoder framework is the use of neural networks known as *recognition networks* or *encoders* to map observed data to the parameters of the variational distributions [19]. Following [20], we use a Gaussian posterior for $\mathbf{z}_i$ and a Log-Normal posterior for l_i, allowing for closed-form solutions for the KL terms. Making use of a specific sampling scheme known as the reparameterization trick [19], we can train both prior and variational parameters in an end-to-end fashion.

2.2 Practical considerations

We implement all neural networks using batch normalization [21] and ReLU activation functions between hidden layers. We use ADAM [22] for iterative stochastic optimization and approximate the expectation in eq. (24) using 5 Monte-Carlo samples from the posterior distribution. To avoid over-regularization at the early stages of training, we apply a scaling factor to the KL term to modulate its influence. More specifically, we found that shrinking the KL term in epoch i by a factor of $\frac{i}{25}$ worked well on our data.

Hyper-parameter for the sci-ATAC-seq data of developing *Drosophila melanogaster* embryos were tuned by maximizing the held-out log likelihood on 20% of the cells. The final choices are shown in table 1.

Table 1: VAE hyper-parameters

Parameter	Value
Cell state dimension k	8
Hidden layers for the encoder $q(\mathbf{z}_i \mid \mathbf{x}_i, c_i)$	[256, 128]
Hidden layers for the encoder $q(l_i, \mid \mathbf{x}_i, c_i)$	[256]
Hidden layers for the decoder $f_\rho(\mathbf{z}_i, c_i)$	[64, 128]
Hidden layers for time module $f_z(\mathbf{z}_i)$	None

References

[1] Swann Floc'hlay, Emily S Wong, Bingqing Zhao, Rebecca R Viales, Morgane Thomas-Chollier, Denis Thieffry, David A Garfield, and Eileen EM Furlong. Cis-acting variation is common across regulatory layers but is often buffered during embryonic development. *Genome research*, 31(2):211–224, 2021.

[2] Natsuhiko Kumasaka, Andrew J Knights, and Daniel J Gaffney. Fine-mapping cellular qtls with rasqual and atac-seq. *Nature genetics*, 48(2):206–213, 2016.

[3] Bryce Van De Geijn, Graham McVicker, Yoav Gilad, and Jonathan K Pritchard. Wasp: allele-specific software for robust molecular quantitative trait locus discovery. *Nature methods*, 12(11):1061–1063, 2015.

[4] Wei Sun. A statistical framework for eqtl mapping using rna-seq data. *Biometrics*, 68(1):1–11, 2012.

[5] Thomas Minka. Estimating a dirichlet distribution, 2000.

[6] Christopher KI Williams and Carl Edward Rasmussen. *Gaussian processes for machine learning*, volume 2. MIT press Cambridge, MA, 2006.

[7] Valentine Svensson, Sarah A Teichmann, and Oliver Stegle. Spatialde: identification of spatially variable genes. *Nature methods*, 15(5):343–346, 2018.

[8] Xihong Lin. Variance component testing in generalised linear models with random effects. *Biometrika*, 84(2):309–326, 1997.

[9] Daowen Zhang and Xihong Lin. Hypothesis testing in semiparametric additive mixed models. *Biostatistics*, 4(1):57–74, 2003.

[10] Seunggeun Lee, Mary J Emond, Michael J Bamshad, Kathleen C Barnes, Mark J Rieder, Deborah A Nickerson, ESP Lung Project Team, David C Christiani, Mark M Wurfel, Xihong Lin, et al. Optimal unified approach for rare-variant association testing with application to small-sample case-control whole-exome sequencing studies. *The American Journal of Human Genetics*, 91(2):224–237, 2012.

[11] Rachel Moore, Francesco Paolo Casale, Marc Jan Bonder, Danilo Horta, Lude Franke, Inês Barroso, and Oliver Stegle. A linear mixed-model approach to study multivariate gene–environment interactions. *Nature genetics*, 51(1):180–186, 2019.

[12] Christoph Lippert, Jing Xiang, Danilo Horta, Christian Widmer, Carl Kadie, David Heckerman, and Jennifer Listgarten. Greater power and computational efficiency for kernel-based association testing of sets of genetic variants. *Bioinformatics*, 30(22):3206–3214, 2014.

[13] Kaare Brandt Petersen and Michael Syskind Pedersen. The matrix cookbook.

[14] Robert B Davies. Algorithm as 155: The distribution of a linear combination of χ 2 random variables. *Journal of the Royal Statistical Society. Series C (Applied Statistics)*, 29(3):323–333, 1980.

[15] Pierre Duchesne and Pierre Lafaye De Micheaux. Computing the distribution of quadratic forms: Further comparisons between the liu–tang–zhang approximation and exact methods. *Computational Statistics & Data Analysis*, 54(4):858–862, 2010.

[16] Huan Liu, Yongqiang Tang, and Hao Helen Zhang. A new chi-square approximation to the distribution of non-negative definite quadratic forms in non-central normal variables. *Computational Statistics & Data Analysis*, 53(4):853–856, 2009.

[17] Samuel S Wilks. The large-sample distribution of the likelihood ratio for testing composite hypotheses. *The annals of mathematical statistics*, 9(1):60–62, 1938.

[18] Carl Doersch. Tutorial on variational autoencoders. *arXiv preprint arXiv:1606.05908*, 2016.

[19] Diederik P Kingma and Max Welling. Auto-encoding variational bayes. *arXiv preprint arXiv:1312.6114*, 2013.

[20] Romain Lopez, Jeffrey Regier, Michael B Cole, Michael I Jordan, and Nir Yosef. Deep generative modeling for single-cell transcriptomics. *Nature methods*, 15(12):1053–1058, 2018.

[21] Sergey Ioffe and Christian Szegedy. Batch normalization: Accelerating deep network training by reducing internal covariate shift. In *International conference on machine learning*, pages 448–456. PMLR, 2015.

[22] Diederik P Kingma and Jimmy Ba. Adam: A method for stochastic optimization. *arXiv preprint arXiv:1412.6980*, 2014.
