# Supplementary material for: scDALI: modeling allelic heterogeneity in single cells reveals context-specific genetic regulation
Source: Genome Biol. 2022 Jan 6;23:8. doi: 10.1186/s13059-021-02593-8 (PMC8734213; doi:10.1186/s13059-021-02593-8)
Supplement: Supplementary file 4 — Additional file 4. Review history. [file 13059_2021_2593_MOESM4_ESM.docx]

Review History

**First round of review**

**Reviewer 1**

**Were you able to assess all statistics in the manuscript, including the appropriateness of statistical tests used?**

No

**Were you able to directly test the methods?**

Yes

**Comments to author:**

In this manuscript, Heinen and colleagues present scDALI, a workflow and probabilistic model that use single cell genomics data to first define cell type and cell state clusters, then use allelic imbalance to identify genetic variants that correlate with molecular measurements (i.e. QTLs). The authors also generated new data in the form of scATAC-seq from multiple developmental stages of F1 Drosophila embryos to further test scDALI. Using scDALI in this dataset, they find several interesting examples in which an eQTL has heterogeneous effects across different cell-types or temporal stages.

Mapping QTLs using single-cell data is becoming increasingly popular, yet there are no established method to do this. Furthermore, there exists no probabilistic framework to identify QTLs that are cell-type or cell-state-specific. Therefore, scDALI seems to be a very timely contribution to the field. That being said, I was disappointed by the lack of benchmarking for QTL calling (see point 3). Here are a few specific comments/suggestions:

1. While I am not an expert on the probabilistic modeling, the choice from the authors to model the data as they did (using a Gaussian Process with a Beta Binomial) appears sensible to me. I particularly like that covariates can be added to the model naturally.

2. In the simulation, it was unclear to me what is the proportion of variance explained by homogeneous/hetergeneous effect relative to all variance?

3. In the iPSC analysis, the authors started with 3,966 eQTL identified in the primary study, but I was left wondering how many of these could be identified using scDALI if one were to do a QTL mapping from scratch using scDALI. More generally, the manuscript does not give a sense for how good scDALI can be used to identify QTLs using single cell genomics data. I do appreciate that the focus is on identifying and distinguishing homogeneous vs heterogeneous effects, but the abstract, introduction and conclusion all put an emphasis on scDALI's ability to identify QTLs, which I don't think is appropriately benchmarked here.

4. Users would want to have a more detailed documentation of both the "tempo" and "scDALI" method, and how they are ran.

5. Related to the above point, the manuscript does not describe how the allelic and total counts tables were generated in sufficient detail. The author should specify the tool used to do this or even include a helper script as part of the scDALI package. This is important because this tool is the first of its kind in the field of scATAC data analysis and users would want a more detailed guidance.

6. In Figure 2a and 2b, the authors show that including donorID as a covariate in the model is necessary. I was wondering whether including one or more other covariates other than just donorID might also be necessary. Cell numbers could be study- or dataset- specific, e.g. from batch effects during library preparation or sequencing, or other technical factors? It might be helpful to advise users on this.

7. The output from scDALI model seems to be peak-level summary statistics (P-values). Can scDALI output SNP-level statistics? For example, within a 1Kb window tested in the manuscript, there are many cases where more than one heterozygous SNP overlap with a peak. One of the SNPs might affect a TF binding site, therefore it would be more informative to have SNP-level statistics to be able to follow-up with a transcription factor foot-printing analysis.

Minor:

Fig 4a,e,h Black dots indicate cells with observed allelic rates (non-zero allelic total counts). Should it say with no observed allelic rates?

**Reviewer 2**

**Were you able to assess all statistics in the manuscript, including the appropriateness of statistical tests used?**

Yes: The statistics were thorough. We had one question about the t-tests, noted below.

**Were you able to directly test the methods?**

No

**Comments to author:**

The authors propose a new computational framework --- single-cell differential allelic imbalance (scDALI) --- for assessing the relationship between allelic imbalance and cell state/cell type. To start, the authors propose using a single-cell count matrix (RNA transcripts, chromatin accessibility, etc.) for two purposes: 1) To estimate the allelic rates for various regions, and 2) To infer a representation of cell state. They then develop a regression model with the cell-specific allelic rates as the response and the cell state dimensions as the input covariates. A hypothesis testing framework is developed for more rigorous statistical testing of cell-state specific genetic regulation. They test scDALI on simulated data, scRNA-seq data from iPS cell lines, and a homegrown sciATAC-seq dataset from developing fly embryos. They find several interesting associations in the latter two datasets, and they differentiate between allelic imbalance that occurs differentially between cell states (heterogeneous), allelic imbalance that occurs globally across cell states (homogeneous), and allelic imbalance that occurs in both settings (joint).

Overall, the paper is thorough and well-written. The proposed method appears robust and seems promising for studying context-specific genetic regulation. It was hard to understand the goals of the study on the first pass, and the figures did not fully clarify, but after a few additional reads through it became clear.

Major comments

Need for further benchmarking. Given that most of the analyses are done with a (generalized) linear model (see minor point 1 below), the hypothesis test essentially reduces to testing the coefficients of a linear model, which is a well-studied area. Currently, the only competing method that is shown is a one-vs-all t-test for testing for differences between discrete cell state clusters. Further benchmarking should be considered, including an ANOVA-style analysis for jointly testing for differences across all clusters.

Need for testing robustness to definition of cell state. Given that the underlying model is seeking associations between a representation of "cell state" and allelic imbalance, some further discussion of the choice of "cell state" representation and its impact on downstream associations with allelic rate is required. It seems that any significant allelic imbalance that is found must be discussed in the context of the cell state that was fed in as an input to the model. For example, how robust are the findings to different choices of embeddings for cell state?

Clarification of relationship to t-test. Related to point (1), Figure 1d and Supplementary Figure 3b don't seem to agree with each other. In particular, scDALI-Het and the t-test seem to have opposite relationships with discrete/continuous FEV in each of the figures. Clarification is needed here, and further clarification on how the simulated data was generated in general is needed.

Need to identify which cell states are associated with differential allelic imbalance. According to Eq. 24 in the Supplemental Methods, the model only allows for a single heterogeneous variance term \sigma^2_{het}. This implies that the model is testing for differential allelic imbalance across all cell state dimensions jointly, rather than individual differences in each dimension. In the case of discrete cell states (e.g., Leiden clusters), this means that the model cannot report which cluster(s) are driving the differential imbalance. It would be useful to test this with the model, as it's often more useful to know which cell states have differential allelic imbalance, rather than that there exists allelic imbalance at all. In the linear model case, this can be done by simply allowing cell state dimension-specific coefficients (or, equivalently, using a GP with a linear kernel, where the linear kernel has dimension-specific variance hyperparameters).

Discussion of any dependence between total counts and allelic rates. Given that both the allelic counts and the cell state are produced from the same dataset, special care must be taken to avoid contamination between the datasets. It would be useful to have more discussion explaining why carving out these two data matrices from the same underlying dataset is statistically reasonable. In particular, can we assume that the total counts matrix and the cell state matrix will be "independent" in the sense that there won't be an artifactual association between them, given the way they're created? Any empirical/analytical evidence to support this would be useful as well.

Minor comments

It's not clear why the authors present the model in a GP framework when they only use linear kernels in their analyses. It might be more straightforward to simply describe it as a linear model, while noting that it can be extended to a nonlinear GP.

The focus on "donor ID" in the analysis of iPS cells is a bit distracting. It seems to me that accounting for donor ID is more of a technical validation, and I would recommend pushing it to the supplement. Instead, it might be worth focusing more on novel discoveries using scDALI in this section.

In eq. 24 in the Methods, it seems like r_i should be \mathbf{r} (the vector of observed allelic rates, rather than a single allelic rate.

**Authors Response**

**Point-by-point responses to the reviewers’ comments:**

Reviewer #1:

In this manuscript, Heinen and colleagues present scDALI, a workflow and probabilistic model that use single cell genomics data to first define cell type and cell state clusters, then use allelic imbalance to identify genetic variants that correlate with molecular measurements (i.e. QTLs). The authors also generated new data in the form of scATAC-seq from multiple developmental stages of F1 Drosophila embryos to further test scDALI. Using scDALI in this dataset, they find several interesting examples in which an eQTL has heterogeneous effects across different cell-types or temporal stages.

Mapping QTLs using single-cell data is becoming increasingly popular, yet there are no established method to do this. Furthermore, there exists no probabilistic framework to identify QTLs that are cell-type or cell-state-specific. Therefore, scDALI seems to be a very timely contribution to the field. That being said, I was disappointed by the lack of benchmarking for QTL calling (see point 3). Here are a few specific comments/suggestions:

*Response: Thank you for the comments. Before addressing the individual points, we would like to mention that part of the concerns raised may also reflect a misunderstanding concerning the scope of scDALI. While the method can be applied to population-scale datasets (as illustrated using human iPSC data), the key use case is to model regulatory variation in a small sample. The model is not designed to identify novel eQTL but instead is focused on interpreting the context-specific nature of regulatory effects. The reason for this is twofold: (i) for smaller sample sizes there is no sufficient genetic variation to fine-map eQTL variants, hence the focus on modelling heterogeneity in allelic imbalance, (ii) even for population-scale studies using single-cell readouts, the primary interest is the characterization of known loci and not discovery of novel effects. The latter point reflects differences in power to detect eQTL in bulk versus single-cell data [1] and the sample sizes that can currently be profiled using single-cell readouts.*

*The annotation of known eQTL in terms of their context specificity is conceptually related to established principles from the analysis of genotype-environment interactions (GxE), where commonly for discovery the focus is on the G component only (eg. ref [2–5]). While we originally addressed this point in the discussion section, we realize that it deserves further clarification. We have revised the main text to more clearly state the purpose and scope of the model and the focus on allelic imbalance. Specifically, see page 5, lines 111-114 and page 21, lines 459-471.*

Major comments

While I am not an expert on the probabilistic modeling, the choice from the authors to model the data as they did (using a Gaussian Process with a Beta Binomial) appears sensible to me. I particularly like that covariates can be added to the model naturally.

In the simulation, it was unclear to me what is the proportion of variance explained by homogeneous/hetergeneous effect relative to all variance?

*Response: It is correct that this parameter is not explicitly given in our simulation experiments. This is because the non-Gaussian noise model prevents us from decomposing noise and signal variance in a straightforward manner. To provide this information we have instead specified the kernel scaling parameter (i.e. the effect size of allelic imbalance) and the likelihood dispersion parameter, which captures the degree of extra-binomial variance (denoted as sigma and theta in Supp. Fig. 1 & 3∫; see also Methods, page 32-34).*

In the iPSC analysis, the authors started with 3,966 eQTL identified in the primary study, but I was left wondering how many of these could be identified using scDALI if one were to do a QTL mapping from scratch using scDALI. More generally, the manuscript does not give a sense for how good scDALI can be used to identify QTLs using single cell genomics data. I do appreciate that the focus is on identifying and distinguishing homogeneous vs heterogeneous effects, but the abstract, introduction and conclusion all put an emphasis on scDALI's ability to identify QTLs, which I don't think is appropriately benchmarked here.

*Response: We thank the reviewer for raising this important point. As already pointed out above, scDALI is specifically designed to characterize regulatory variation using allelic imbalance. We have revised the text to further clarify the purpose and scope of the model and to motivate the focus on modelling allelic imbalance. We also note that we have restructured the manuscript and now present the human iPS experiments at the end of the paper, which also helps to clarify the focus and the framing of the method (previously Fig. 2 now Fig. 5).*

*Having said that, we of course agree that benchmarking of any novel method is of course important. In response to comments raised by R2 comment 1, we have extended our simulation study and now consider additional baseline models (ANOVA & a linear model fixed-effect likelihood ratio test), and we have extended the benchmarking settings.*

Users would want to have a more detailed documentation of both the "tempo" and "scDALI" method, and how they are ran.

*Thank you. We have substantially revised and extended the software documentation: https://pmbio.github.io/scdali. In particular, we now provide detailed information on the usage of the software and we provide recommendations for the definition of cell states based on different input data modalities. We also provide guidance on how to account for confounding factors in the analysis.*

Related to the above point, the manuscript does not describe how the allelic and total counts tables were generated in sufficient detail. The author should specify the tool used to do this or even include a helper script as part of the scDALI package. This is important because this tool is the first of its kind in the field of scATAC data analysis and users would want a more detailed guidance.

*Response: The processing of the scATAC data followed the published pipeline of Cusanovich et al. (https://github.com/shendurelab/fly-atac/). In response to this comment, we have included additional details in Methods and we extended the section on the quantification of allele-specific counts. Additionally, the specific quantification script we used is now accessible at https://github.com/tohein/scai_utils and referenced in the scDALI documentation. We also provide further details in Methods concerning specific processing steps and the approaches that can be used and what is important for running scDALI (pages 24 & 25). For example, if the focus is on detecting heterogeneity rather than detecting total AI, reference biases may be less critical than in applications where the total AI is of interest. Many of these choices will depend on the goals of a study. We provide more details for the users on the scDALI website https://pmbio.github.io/scdali.*

In Figure 2a and 2b, the authors show that including donorID as a covariate in the model is necessary. I was wondering whether including one or more other covariates other than just donorID might also be necessary. Cell numbers could be study- or dataset- specific, e.g. from batch effects during library preparation or sequencing, or other technical factors? It might be helpful to advise users on this.

*Response: There is an argument that the analysis of allelic variation is less affected by technical factors and batch effects than the total expression levels. In particular if both alleles are affected similarly by technical effects, these cancel out when quantifying allelic differences. Only confounding factors that affect the allelic rate do require attention in scDALI. Having said this, it is of course advisable to adjust for additional technical confounders (batch effects, size factors) when constructing the cell-state representation, which is based on typical counts. We now provide additional guidance in Methods (pages 24 & 25).*

*Response: In the multi-individual study presented in Fig. 5, we aggregated cells from multiple individuals using phasing to relate allelic rates relative to the genotypic state of a known eQTL variant. In this setting, differences between individuals and batch could cause cells from different individuals to be distributed non-uniformly across different cell states. The reasons for this could for example be (i) genetic variation in the eGene, potentially leading to individual-specific mapping biases when quantifying allele-specific counts or (ii) unaccounted cell-state-specific eQTL variants that are different from the lead eQTL of interest. Consequently, we have included the donorID as covariates, which achieves the desired calibration.*

The output from scDALI model seems to be peak-level summary statistics (P-values). Can scDALI output SNP-level statistics? For example, within a 1Kb window tested in the manuscript, there are many cases where more than one heterozygous SNP overlap with a peak. One of the SNPs might affect a TF binding site, therefore it would be more informative to have SNP-level statistics to be able to follow-up with a transcription factor foot-printing analysis.

*Response: The reviewer is correct. This point is related to the question whether scDALI can be used to identify causal SNPs and/or the resolution at which we can identify AI. Please see our answer above regarding the purpose of the model. We apologise if this was not clear in our previous version of the manuscript. We have now also extended the Discussion to better reflect this (page 21, paragraph 2). Having said this, if a sufficiently large set of different individuals is available, it is possible to apply scDALI using phased data relative to different candidate variants. This approach could in principle be used to assess the statistical evidence for cell-state-specific effects across a range of alternative variants. Given that the focus of scDALI is the analysis of a small sample, we have not considered this further.*

Minor comments

Fig 4a,e,h Black dots indicate cells with observed allelic rates (non-zero allelic total counts). Should it say with no observed allelic rates?

*Response: Black dots indicate cells where at least one read overlapped the given peak, thus allowing the quantification of allelic imbalance. That is, these cells were used as data points to fit the scDALI model for this peak. To visualize allelic imbalance in the remaining cells, we then predicted allelic rates using their cell-state coordinates. We have updated the legend for Fig. 3 (formerly Fig. 4) to clarify this point.*

Reviewer #2:

The authors propose a new computational framework --- single-cell differential allelic imbalance (scDALI) --- for assessing the relationship between allelic imbalance and cell state/cell type. To start, the authors propose using a single-cell count matrix (RNA transcripts, chromatin accessibility, etc.) for two purposes: 1) To estimate the allelic rates for various regions, and 2) To infer a representation of cell state. They then develop a regression model with the cell-specific allelic rates as the response and the cell state dimensions as the input covariates. A hypothesis testing framework is developed for more rigorous statistical testing of cell-state specific genetic regulation. They test scDALI on simulated data, scRNA-seq data from iPS cell lines, and a homegrown sciATAC-seq dataset from developing fly embryos. They find several interesting associations in the latter two datasets, and they differentiate between allelic imbalance that occurs

differentially between cell states (heterogeneous), allelic imbalance that occurs globally across cell states (homogeneous), and allelic imbalance that occurs in both settings (joint).

Overall, the paper is thorough and well-written. The proposed method appears robust and seems promising for studying context-specific genetic regulation. It was hard to understand the goals of the study on the first pass, and the figures did not fully clarify, but after a few additional reads through it became clear.

*Response: We thank the reviewer for this assessment. We would like to apologize for the confusion and lack of clarity in parts of the manuscript. In response to this comment and similar concerns raised by R1, we have restructured the manuscript and substantially revised the main text to improve clarity and the motivation of the model and its intended use.*

Major comments

Need for further benchmarking. Given that most of the analyses are done with a (generalized) linear model (see minor point 1 below), the hypothesis test essentially reduces to testing the coefficients of a linear model, which is a well-studied area. Currently, the only competing method that is shown is a one-vs-all t-test for testing for differences between discrete cell state clusters. Further benchmarking should be considered, including an ANOVA-style analysis for jointly testing for differences across all clusters.

*Response: Thank you for highlighting this point. In response to this comment, we have updated the simulation study and replaced the one-vs-all t-test with an ANOVA analysis as suggested. The ANOVA model is indeed a stronger baseline, but we feel that the advantages offered by scDALI remain substantial.*

*Additionally, we have also considered further baseline approaches based on a linear model that explains cell-state-specific effects using a multiple degrees of freedom test (likelihood ratio). Consistent with results reported in Moore et al. 2019, we observe that such a fixed effect test can lead to inflated p-values. This effect is most prominent in the regime of larger numbers of cell-state covariates, which is exactly the regime of interest for using scDALI. In contrast, the proposed kernel-based score test (scDALI-Het) maintains calibration across a wide range of settings (updated Supp. Fig. 1 & 3).*

Need for testing robustness to definition of cell state. Given that the underlying model is seeking associations between a representation of "cell state" and allelic imbalance, some further discussion of the choice of "cell state" representation and its impact on downstream associations with allelic rate is required. It seems that any significant allelic imbalance that is found must be discussed in the context of the cell state that was fed in as an input to the model. For example, how robust are the findings to different choices of embeddings for cell state?

*Response: In principle, both the power and the interpretation of the identified GxC effects will depend on the specific cell-state representation that is used. In general, this representation should be chosen to reflect the underlying cellular heterogeneity, e.g. using a biologically meaningful cell type annotation or (pseudo-)temporal ordering. However, we agree that choosing an appropriate representation for complex data such as developmental studies can be challenging. As a general purpose approach, we propose to use a lower-dimensional embedding that may reflect both continuous and discrete effects. We also provide some guidance in Methods (pages 24 & 25) on how to select a suitable coordinate system to test for cell-state-specific effects.*

*Concerning the question of robustness, we now present additional results when using an alternative cell embedding, comparing the results from our variational autoencoder to latent semantic indexing (LSI) and latent Dirichlet allocation (cisTopic) (Supp. Fig. 10a, b). These results are discussed in the main text (page 10). While there is some variation in statistical significance, the most significant findings robustly replicate across these alternative cell-state representations.*

Clarification of relationship to t-test. Related to point (1), Figure 1d and Supplementary Figure 3b don't seem to agree with each other. In particular, scDALI-Het and the t-test seem to have opposite relationships with discrete/continuous FEV in each of the figures. Clarification is needed here, and further clarification on how the simulated data was generated in general is needed.

*Response: We apologize for this oversight. The figures were labelled incorrectly, which explains the counterintuitive trends. In response to comment 1, we have also replaced the t-test with a one-way ANOVA model (see above) and updated the associated Supp. Fig. 1 & 3. The results shown are now labelled correctly and agree with the main figure. Additionally we extended Methods to provide more details on the scheme for the generation of simulated data, covering calibration experiments for different additional baseline models as well as power analyses. These updated results are now discussed at page 32, Evaluation of scDALI on simulated data.*

Need to identify which cell states are associated with differential allelic imbalance. According to Eq. 24 in the Supplemental Methods, the model only allows for a single heterogeneous variance term \sigma^2_{het}. This implies that the model is testing for differential allelic imbalance across all cell state dimensions jointly, rather than individual differences in each dimension. In the case of discrete cell states (e.g., Leiden clusters), this means that the model cannot report which cluster(s) are driving the differential imbalance. It would be useful to test this with the model, as it's often more useful to know which cell states have differential allelic imbalance, rather than that there exists allelic imbalance at all. In the linear model case, this can be done by simply allowing cell state dimension-specific coefficients (or, equivalently, using a GP with a linear kernel, where the linear kernel has dimension-specific variance hyperparameters).

*Response: Indeed, scDALI-Het is based on an omnibus test for detecting heterogeneous allelic imbalance and as such does not test for associations with individual cell-state components. In fact, the entire motivation of our model is to mitigate the need to define individual cell state covariates and instead define a joint test for the effect of the cell state manifold as a whole on allelic imbalance.*

*Having said this, we agree that the ability to interpret the allele-specific effects identified by scDALI will be important. While estimating effect sizes that are specific to individual dimensions would be possible within our framework, we argue that interpreting individual cell-state components is often challenging, in particular when using non-linear embeddings with no clear peak / gene association.*

*As an alternative, we now report and describe two downstream analyses to address this. The first option is to break down the variation on the level of well-characterized clusters such as known lineages or cell types. Results from this approach were previously described in Figure 4c, g, j, now Supp. Fig. 11b-d. Additionally, we now consider a strategy that allows to avoid any (even downstream) discretization of the cell state manifold. Instead, we propose to compare estimated allelic rates with cell-level scores of transcription factor (TF) or pathway activity. As an example, we applied chromVAR to assess the activity of 65 TFs based on openness of associated motives in the Drosophila F1 data and ranked these TFs based on the correlation of their activity with estimated allelic rates across cells. The results from this approach are now presented in Figure 3c, g, j, and discussed on pages 11-13.*

Discussion of any dependence between total counts and allelic rates. Given that both the allelic counts and the cell state are produced from the same dataset, special care must be taken to avoid contamination between the datasets. It would be useful to have more discussion explaining why carving out these two data matrices from the same underlying dataset is statistically reasonable. In particular, can we assume that the total counts matrix and the cell state matrix will be "independent" in the sense that there won't be an artifactual association between them, given the way they're created? Any empirical/analytical evidence to support this would be useful as well.

*Response: Thank you for pointing this out; it is indeed important to discuss possible dependencies between allelic rates and total counts and possible false positive results. It is conceivable that the presence of allelic imbalance could lead to a reduction in total counts for a particular gene / peak, thereby influencing the cell state representation and producing artifactual associations. On the other hand, it is arguably expected that a change in cell state could affect both the total read count and the allelic rate.*

*To address this potential concern, we performed an association analysis, testing for dependencies between total and allele-specific counts per peak (Supp. Fig. 10c, d), which shows that for the vast majority of the peaks with GxC effects no such correlations are observed.*

Minor comments

It's not clear why the authors present the model in a GP framework when they only use linear kernels in their analyses. It might be more straightforward to simply describe it as a linear model, while noting that it can be extended to a nonlinear GP.

*Response: We apologize for the confusion. We have updated the nomenclature and now describe scDALI as an instance of a generalized linear mixed model.*

The focus on "donor ID" in the analysis of iPS cells is a bit distracting. It seems to me that accounting for donor ID is more of a technical validation, and I would recommend pushing it to the supplement. Instead, it might be worth focusing more on novel discoveries using scDALI in this section.

*Response: We believe that accounting for donor IDs is perhaps a subtle but important aspect of the application of scDALI to population-scale data. By incorporating donor IDs as covariates in the scDALI model, we can account for donor-specific reference mapping biases in order to reduce false positive results. This is contrary to the analysis of heterogeneous allelic imbalance within a single sample where mapping biases can be neglected.*

In eq. 24 in the Methods, it seems like r_i should be \mathbf{r} (the vector of observed allelic rates, rather than a single allelic rate.

*Response: Thank you for pointing this out, we have updated the equation.*

*References*

*1. Cuomo ASE, Alvari G, Azodi CB, single-cell eQTLGen consortium, McCarthy DJ, Bonder MJ. Optimizing expression quantitative trait locus mapping workflows for single-cell studies. Genome Biol. 2021;22:188.*

*2. Moore R, BIOS Consortium, Casale FP, Bonder MJ, Horta D, Franke L, et al. A linear mixed-model approach to study multivariate gene–environment interactions [Internet]. Nature Genetics. 2019. p. 180–6. Available from: http://dx.doi.org/10.1038/s41588-018-0271-0*

*3. Casale FP, Horta D, Rakitsch B, Stegle O. Joint genetic analysis using variant sets reveals polygenic gene-context interactions. PLoS Genet. 2017;13:e1006693.*

*4. Kraft P, Yen Y-C, Stram DO, Morrison J, Gauderman WJ. Exploiting gene-environment interaction to detect genetic associations. Hum Hered. 2007;63:111–9.*

*5. Lin X, Lee S, Christiani DC, Lin X. Test for interactions between a genetic marker set and environment in generalized linear models. Biostatistics. 2013;14:667–81.*

**Second round of review**

**Reviewer 1**

I would like to thank the authors for the clarifications and addressing my comments.

**Reviewer 2**

Thank you to the authors for their thorough response to the initial review. They have addressed nearly all comments to a satisfactory extent, and I only have a few minor remaining comments below. Once these comments are addressed, I believe the paper is fit for publication.

I'm still a bit puzzled with one aspect of the comparison to the ANOVA approach. On the left end of Fig. 1d, you test the models using a simulated dataset made up of 100% continuous cell state covariates. How are you running an ANOVA in this case? Randomly assigning groups? In this case, it might make more sense to make a comparison against a method that is more appropriate for this continuous case (such as testing whether the coefficients of a linear model are zero, although this is basically equivalent to scDALI).

How are you computing power (e.g., in Fig. 1c, d)? Please include the exact steps, perhaps in the supplement. In addition, rather than solely examining power, shouldn't you be looking at both sensitivity and specificity of the test, not just sensitivity? If not, it may be worth explaining why.

**Authors Response**

**Point-by-point responses to the reviewers’ comments:**

Reviewer #2: Thank you to the authors for their thorough response to the initial review. They have addressed nearly all comments to a satisfactory extent, and I only have a few minor remaining comments below. Once these comments are addressed, I believe the paper is fit for publication.

I'm still a bit puzzled with one aspect of the comparison to the ANOVA approach. On the left end of Fig. 1d, you test the models using a simulated dataset made up of 100% continuous cell state covariates. How are you running an ANOVA in this case? Randomly assigning groups? In this case, it might make more sense to make a comparison against a method that is more appropriate for this continuous case (such as testing whether the coefficients of a linear model are zero, although this is basically equivalent to scDALI).

*We apologize for the lack of clarity. In Fig. 1d, we vary the approach taken to simulate allelic effects, while retaining the exact same approach for testing. Briefly, ‘continuous’ refers to allelic imbalance drawn from the VAE representation (i.e. coordinates in the VAE latent space) whereas ‘discrete’ refers to a simulated settings where the Leiden clustering of cells based on their distance in the VAE space is used to simulated allelic imbalance. The simulated scenarios consider different interpolations between these two extreme settings. For each simulated scenario, the ANOVA test identifies differences between the Leiden clusters (the discrete representation), while scDALI uses the VAE (continuous) representation. We have clarified this in the description of the simulation experiments in the main text (page 7, paragraph 1).*

How are you computing power (e.g., in Fig. 1c, d)? Please include the exact steps, perhaps in the supplement. In addition, rather than solely examining power, shouldn't you be looking at both sensitivity and specificity of the test, not just sensitivity? If not, it may be worth explaining why.

*Thank you for pointing this out. Power was evaluated as the fraction of simulated regions with allelic imbalance that could be identified at a significance level of alpha=0.05. This is now clarified in Methods (page 34). It is correct that we did not assess specificity in the simulated settings used for the power analysis. Instead, we verified the statistical calibration of all three scDALI tests using data simulated from their corresponding null models (Supp. Fig. 1b, c), showing that the p-value distribution is approximately uniform. This means that the scDALI tests allow for controlling the probability of falsely rejecting the null hypothesis (no imbalance) for any given significance level alpha.*
